# Acid-Activated Motion Switching of DB24C8 between Two Discrete Platinum(II) Metallacycles

**DOI:** 10.3390/molecules26030716

**Published:** 2021-01-30

**Authors:** Yi-Xiong Hu, Gui-Yuan Wu, Xu-Qing Wang, Guang-Qiang Yin, Chang-Wei Zhang, Xiaopeng Li, Lin Xu, Hai-Bo Yang

**Affiliations:** 1 Shanghai Key Laboratory of Green Chemistry and Chemical Processes, School of Chemistry and Molecular Engineering, East China Normal University, 3663 N. Zhongshan Road, Shanghai 200062, China; 52184300024@stu.ecnu.edu.cn (Y.-X.H.); wgy@ahnu.edu.cn (G.-Y.W.); xqwang@chem.ecnu.edu.cn (X.-Q.W.); cwzhang@chem.ecnu.edu.cn (C.-W.Z.); 2 Anhui Province Key Laboratory of Optoelectronic Material Science and Technology, School of Physics and Electronic Information, Anhui Normal University, Wuhu 241002, China; 3 College of Chemistry and Environmental Engineering, Shenzhen University, Shenzhen 518055, China; 13162729689@163.com (G.-Q.Y.); xiaopengli@szu.edu.cn (X.L.)

**Keywords:** pseudorotaxanes, metallacycle, self-sorting, host–guest interaction, molecular motion

## Abstract

The precise operation of molecular motion for constructing complicated mechanically interlocked molecules has received considerable attention and is still an energetic field of supramolecular chemistry. Herein, we reported the construction of two tris[2]pseudorotaxanes metallacycles with acid–base controllable molecular motion through self-sorting strategy and host–guest interaction. Firstly, two hexagonal Pt(II) metallacycles **M1** and **M2** decorated with different host–guest recognition sites have been constructed via coordination-driven self-assembly strategy. The binding of metallacycles **M1** and **M2** with dibenzo-24-crown-8 (DB24C8) to form tris[2]pseudorotaxanes complexes **TPRM1** and **TPRM2** have been investigated. Furthermore, by taking advantage of the strong binding affinity between the protonated metallacycle **M2** and DB24C8, the addition of trifluoroacetic acid (TFA) as a stimulus successfully induces an acid-activated motion switching of DB24C8 between the discrete metallacycles **M1** and **M2**. This research not only affords a highly efficient way to construct stimuli-responsive smart supramolecular systems but also offers prospects for precisely control multicomponent cooperative motion.

## 1. Introduction

Molecular machines in living systems have long been in widespread use and played a critical role in life’s activities [1,2]. Inspired by this, chemists have devoted time to designing, synthesizing and assembling molecules in laboratory conditions to obtain man-made molecular machines with amusing processes and attractive functions [3,4,5,6,7]. As a result, during the past few decades, varieties of man-made molecular machines such as molecular pumps [8,9], molecular muscles [10,11,12,13], molecular shuttles [14,15,16,17], and others [18,19,20] have been successfully fabricated. Mechanically interlocked molecules (MIMs) [21,22], with intriguing topologies and unique dynamic features, have been considered as a versatile platform in developing molecular machines.

Pseudorotaxanes, a type of MIMs which consist of axles encircled by ring components, have received widespread attention in the fields of supramolecular chemistry and materials science [23,24,25,26,27]. Up to now, the most common strategy to prepare pseudorotaxanes is to use macrocylic hosts including crown ethers, cyclodextrins, calixarenes, pillararenes, cucurbiturils etc. encircling the linear guest molecules [28,29,30,31,32,33,34]. In addition, it is worth noting that pseudorotaxanes were usually used as precursors toward the construction of rotaxanes, catenanes and molecular switches [35,36,37,38,39,40,41,42]. As a consequence, the design and preparation of pseudorotaxanes, especially with intriguing and novel topologies, have been raising more interest and are still greatly challenging.

The coordination-driven self-assembly strategy is one of the major developments in supramolecular chemistry during the past few decades [43,44]. To date, by taking advantage of highly directional and relatively strong metal–ligand bonds, massive numbers of discrete supramolecular coordination complexes (SCCs) including two-dimensional (2-D) metallacycles and three-dimensional (3-D) metallacages have been prepared [45,46,47,48]. The constructed supramolecular coordination architectures have shown aesthetical features with well-defined shapes, sizes, and geometries and enabled a range of functions including sensing, drug delivery, liquid crystal, control of optical properties, and so on [49,50,51,52,53,54,55,56,57,58,59]. For example, our group have reported the construction of complex multicomponent systems based on metallacycles through orthogonal self-assembly and the self-sorting strategy, in which eighteen precursors could efficiently self-assemble into two discrete tris[2]pseudorotaxanes in one pot [60]. Due to the straightforward and efficient preparation method, the fabrication of novel pseudorotaxane systems based on SCCs assumes outstanding advantage and offers a great possibility to achieve cooperative motion.

**Scheme 1:**
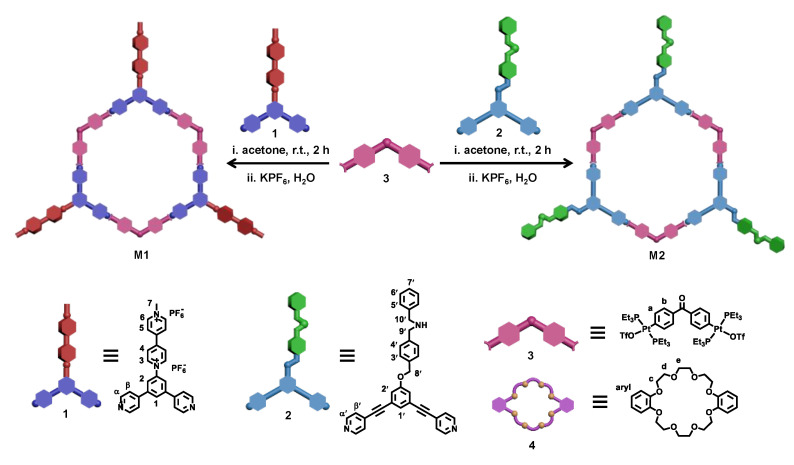
Self-assembly of 120° donor ligand **1** or **2** and the 120° diplatinum (II) acceptor **3** into discrete hexagonal metallacycle **M1** or **M2**.

Toward this goal, herein, we designed and synthesized two 120° dipyridyl donors (**1** and **2**) decorated with bipyridinium (BIPY^2+^) and dibenzylamine (R_1_NHR_2_), respectively (Scheme 1). When the 120° diplatinum(II) acceptor was combined with different 120° dipyridyl donors, two hexagonal Pt(II) metallacycles modified by bipyridinium (BIPY^2+^) or dibenzylamine (R_1_NHR_2_) on the periphery were successfully formed through coordination-driven self-assembly. In addition, due to the difference of molecular size between two 120° dipyridyl donors, the prepared metallacycles could coexist in one pot and exhibited great self-sorting properties. Furthermore, DB24C8 was carefully employed to bind different metallacycles to form tris[2] pseudorotaxanes through host–guest interaction. More interestingly, owing to the binding ability of DB24C8 with the protonated recognition sites of R_1_NHR_2_ (R_1_NH_2_^+^R_2_), which is stronger than that of BIPY^2+^ [11], an acid activated process could be implemented to induce motion switching of DB24C8 between discrete metallacycles **M1** and **M2**. This research provides a novel strategy to fabricate multicomponent cooperative motion switching in complex systems and exhibits the potential for constructing stimuli-responsive smart materials.

## 2. Results and Discussion

### 2.1. Synthesis and Characterization

**Figure 1:**
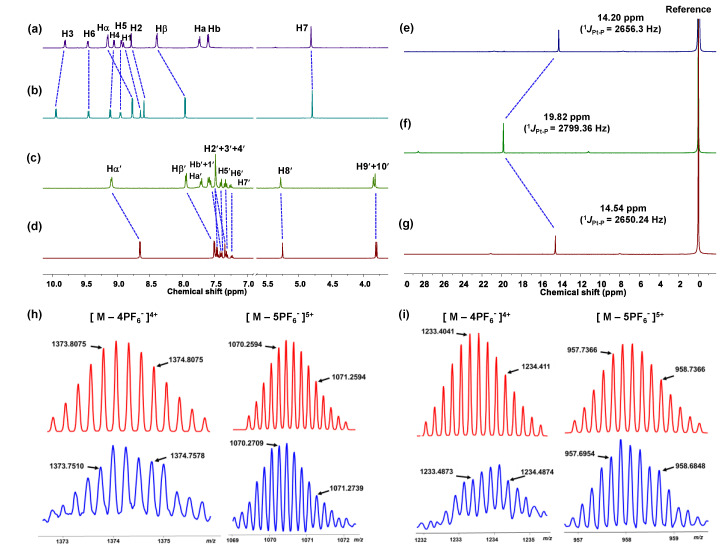
Partial ^1^H NMR spectra (500 M, *d*-acetone, 298 K) of (**a**) metallacycle **M1**, (**b**) ligand **1**, (**c**) metallacycle **M2**, (**d**) ligand **2**. ^31^P NMR spectra (202 MHz, 298 K) of (**e**) metallacycle **M1** (*d*-acetone), (**f**) 120° acceptor **3** (CD_2_Cl_2_), (**g**) metallacycle **M2** (*d*-acetone). Theoretical (red) and experimental (blue) electrospray ionization time-of-flight mass spectrometry (ESI-TOF-MS) spectra of (**h**) metallacycle **M1** and (**i**) metallacycle **M2**.

The Zinke reaction of compound **S1** with compound **S2** yielded the 120° dipyridyl donor ligand **1** in a moderate yield as shown in Scheme S1. Moreover, the 120° dipyridyl donor ligand **2** was easily synthesized through an esterification reaction between compounds **S3** and **S4** (Scheme S2). Stirring the mixtures of 120° donor ligand **1** or **2** with 120° di-Pt(II) acceptor **3** in the stoichiometric ratio in acetone led to the formation of metallacycle **M1** or **M2** guided by the coordination-driven self-assembly approach, respectively (Scheme 1). Multinuclear NMR ^31^P {^1^H} and ^1^H spectra analysis of the metallacycles **M1** and **M2** revealed the formation of discrete metallacycles with highly symmetric structures (Figure 1). For instance, both the ^31^P {^1^H} NMR spectra of **M1** and **M2** displayed a sharp singlet (ca. 14.20 ppm for **M1** and 14.54 ppm for **M2**). The two singlets have been shifted upfield from the starting 120° di-Pt(II) acceptor **3**, which appears at approximately 19.82 ppm (Figure 1e–g). This change, as well as the decrease in coupling of the flanking ^195^Pt satellites (ca. Δ*J*_PPt_ = −143.06 Hz for **M1** and Δ*J*_PPt_ = −149.12 Hz for **M2**), was consistent with the electron back-donation from the platinum atoms. Moreover, as shown in Figure 1a–d, the ^1^H NMR spectrum of metallacycle **M1** or **M2** displayed the downfield shifts of the pyridyl proton signals compared with the 120° dipyridyl donor **1** or **2** (ca. Δδ = 0.38 or 0.44 ppm of Hα or Hβ for **M1**; Δδ = 0.44 or 0.44 ppm of Hα or Hβ for **M2**). All these phenomena were attributed to the loss of electron density upon coordination by the nitrogen lone pair to platinum metal centers, which indicated the formation of platinum-nitrogen (Pt-N) bonds as well.

Further characterization by two-dimensional (2-D) spectroscopic techniques (^1^H-^1^H COSY, NOESY and DOSY) agreed with the formation of the metallacycles **M1** and **M2** (Figures S1–S6). The results showed that the presence of cross-peaks between the signals of the pyridine protons (α-H and β-H) and the PEt_3_ protons (-CH_2_ and -CH_3_) in the 2-D NOESY spectrum, which supported the formation of Pt-N bonds (Figures S2 and S5). Moreover, 2-D diffusion-ordered spectroscopy (DOSY) was also employed to evaluate the discrete metallacycles **M1** and **M2**. All DOSY spectra of **M1** and **M2** presented one set of signals that indicated the existence of discrete metallacycles **M1** or **M2**, respectively (Figures S3 and S6). Mass spectrometric studies of metallacycles **M1** and **M2** were performed by using electrospray ionization time-of-flight mass spectrometry (ESI-TOF-MS), which provided further strong support for metallacycles **M1** and **M2**. For instance, the ESI-TOF-MS spectrum of **M1** or **M2** revealed peaks at *m*/*z* = 1373.9945, 1070.2709, 1233.9489, 958.2781, corresponding to the [M1−4PF_6_^−^]^4+^, [M1−5PF_6_^−^]^5+^, [M2−4PF_6_^−^]^4+^ and [M2–5PF_6_^−^]^5+^ species, respectively. These peaks were isotopically resolved (Figure 1h–j) and agreed very well with their theoretical distribution.

### 2.2. Self-Sorting Process of Metallacycles **M1** and **M2**

**Figure 2:**
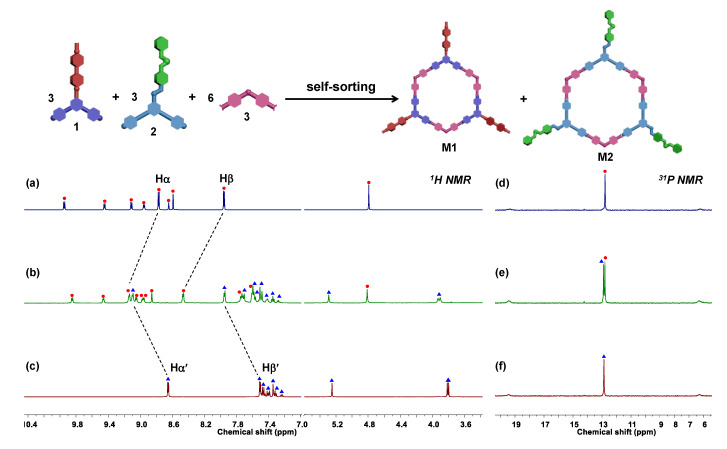
The partial ^1^H NMR spectra (500 MHz, 298 K, *d*-acetone) (left) of the ligand **1** (**a**); the size-controlled self-sorting system (**b**); and the ligand **2** (**c**). The partial ^31^P NMR spectra (202 MHz, 298 K) (right) of the individual metallacycle **M1** (**d**); the size-controlled self-sorting system (**e**); and the individual metallacycle **M2** (**f**).

Upon stirring the mixture of 120° donor ligands **1** and **2** with 120° diplatinum-(II) acceptor **3** in a ratio of 1:1:2 in *d*_6_-acetone at room temperature for 2 h, two discrete metallacycles **M1** and **M2** with different sizes were constructed by the coordination-driven self-assembly and self-sorting one-pot method. The self-sorting process has been demonstrated by ^31^P {^1^H} NMR and ^1^H NMR spectra (Figure 2). For example, two sets of sharp signal peaks were found in ^31^P NMR, 12.88 and 12.79 ppm, respectively, which indicate the successful preparation of two different dominant platinum containing assemblies (Figure 2e). Moreover, as shown in Figure S19, superimposed ^31^P NMR and ^1^H NMR spectra of two individual metallacycles **M1** and **M2** with the self-sorting system highly matched signal peaks, providing further evidence to confirm the self-sorting process. The self-sorting system that was controlled by the size of different 120° donor ligands provided discrete, different sized metallacycles **M1** and **M2** modified with different host–guest recognition sites.

### 2.3. Tris[2]pseudorotaxanes Metallacycles **TPRM1** and **TPRM2**

**Figure 3:**
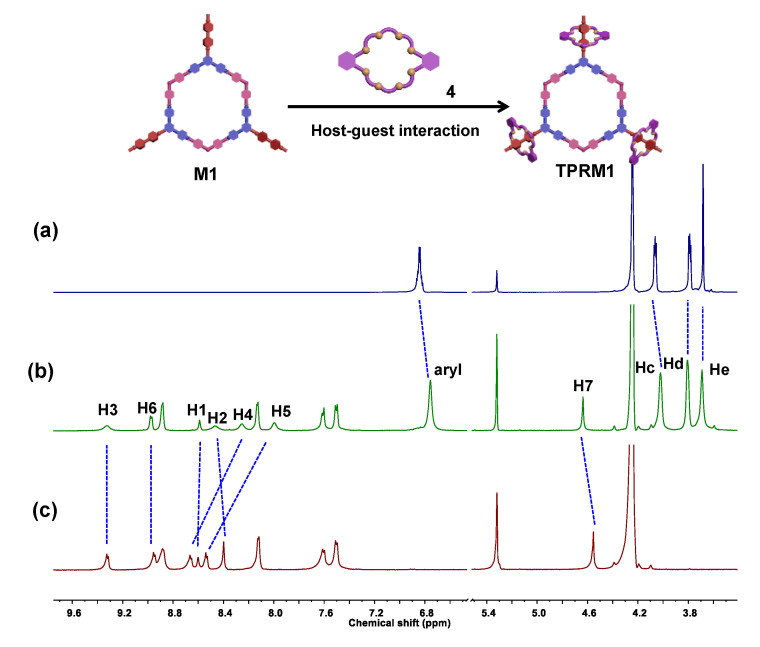
The ^1^H NMR spectra (500 MHz, 298 K) in CD_2_Cl_2_/CD_3_NO_2_ (1:1, *v/v*) of the tris[2]pseudorotaxanes **TPRM1** system (**a**) compound **4**; (**b**) tris[2]pseudorotaxanes **TPRM1**; (**c**) metallacycle **M1**.

**Figure 4:**
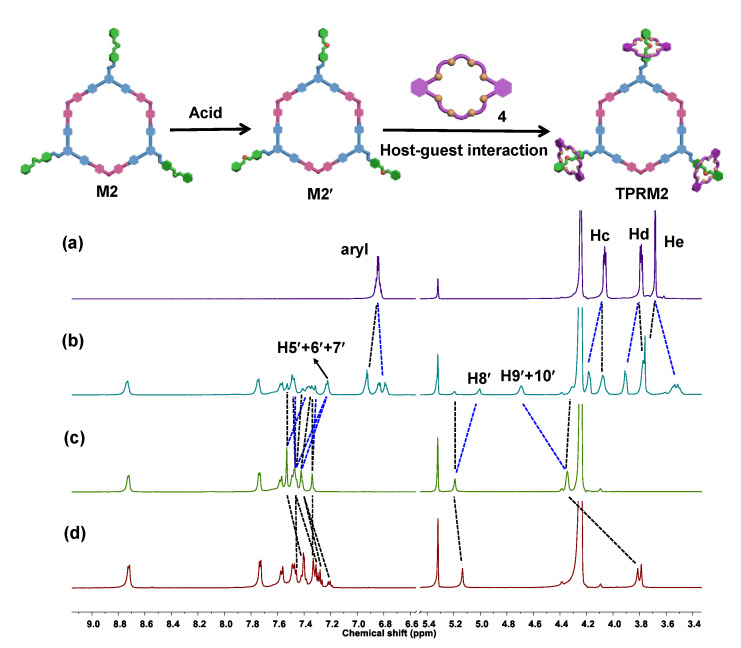
The ^1^H NMR spectra (500 MHz, 298 K) in CD_2_Cl_2_/CD_3_NO_2_ (1:1, *v/v*) of the tris[2]pseudorotaxanes **TPRM2** system (**a**) compound **4**; (**b**) tris[2]pseudorotaxanes **TPRM2**; (**c**) metallacycle **M2′**; (**d**) metallacycle **M2**. (complexed signals of blue line and uncomplexed signals of black line).

The highly efficient coordination-driven self-assembly of multiple recognition sites-containing derivatives provides the possibility to generate higher-order [2]pseudorotaxanes such as tris[2]pseudorotaxanes. Tris-recognition sitescontaining derivatives **M1** or **M2** (BIPY^2+^ units for **M1** and R_1_NHR_2_ units for **M2**) was selected to thread compound **4** (DB24C8) to form two different tris[2]pseudorotaxanes metallacycles **TPRM1** or **TPRM2**, respectively (Figure 3 and Figure 4). The ^1^H NMR spectra of a 1:3 mixtures of metallacycle **M1** and compound **4** in CD_2_Cl_2_/CD_3_NO_2_ (1/1, *v/v*) showed significant shifts due to the complexation of BIPY^2+^ units and compound **4** (Figure 3). For instance, it was found that protons H4 and H5 on BIPY^2+^ units of metallacycle **M1** shifted upfield from 8.67 to 8.25 ppm and 8.54 to 8.00 ppm, respectively, and all proton signals on BIPY^2+^ recognition sites became broad. Moreover, the aryl protons on compound **4** were found to shift upfield from 6.84 to 6.75 ppm. Further characterization with 2-D spectroscopic techniques (^1^H-^1^H COSY and NOESY) proved the formation of tris[2]pseudorotaxanes metallacycle **TPRM1**. For example, the 2-D NOESY spectrum of **TPRM1** clearly exhibited the presence of through-space interactions between the Hc proton of the complexed compound **4** and the BIPY^2+^ protons H6 (Figure S10). All obtained results supported that the metallacycle **M1** with three recognition sites was threaded through the cavity of compound **4** to form host–guest complexation tris[2]pseudorotaxanes metallacycle **TPRM1** in the solution.

As shown in Figure 4, the host–guest interaction of metallacycle **M2** and compound **4** have also been investigated for forming host–guest complexation tris[2]pseudorotaxanes metallacycle **TPRM2** by ^1^H NMR, 2-D spectroscopic techniques (^1^H-^1^H COSY and NOESY). The R_1_NHR_2_ units on the metallacycle **M2** exposed to 3.0 eq. trifluoroacetic acid (TFA) could be protonated and formed the metallacycle **M2’** with three R_1_NH_2_^+^R_2_ units. The protons on R_1_NH_2_^+^R_2_ exhibited downfield shifts (ca. Δ*δ* = 0.55 ppm of H9’ + 10’; Δ*δ* = 0.13 ppm of H3’ + 4’) in the ^1^H NMR spectra of **M2** and 3.0 eq. TFA in a mixed solution of CD_2_Cl_2_/CD_3_NO_2_ (Figure 4c,d). Then the metallacycle **M2’** and compound **4** combined with 1:3 ratio in CD_2_Cl_2_/CD_3_NO_2_ (1/1, *v/v*), as shown in Figure 4b, and the observation of three sets of peaks in the ^1^H NMR spectra was attributed to uncomplexed metallacycle **M2’**, uncomplexed compound **4**, and the complex between metallacycle **M2’** and compound **4**. For example, the partial proton signals of H8’ that take place downfield shifted from 5.19 to 5.00 ppm, and the partial aryl protons of compound **4** also shifted to 6.92 from 6.84 ppm. Notably, the above-mentioned processes also characterized by 2-D ^1^H-^1^H COSY and NOESY (Figures S13–S17), which provided further support for the formation of tris[2]pseudorotaxanes metallacycle **TPRM2** through host–guest interaction with metallacycle **M2’** and compound **4**. As a result, the addition of acid as stimulus endows metallacycle **M2** a switchable feature that controlling the host–guest property via chemical stimulus. In addition, ^1^H NMR titration experiments were investigated for two tris[2]pseudorotaxanes metallacycles **TPRM1** and **TPRM2** (Figures S12 and S18).

### 2.4. Acid-Activated Motion Switching of DB24C8

Owing to the following advantages: (i) different sizes of metallacycles **M1** and **M2** could coexist in one pot via self-sorting strategy; (ii) different recognition sites of two metallacycles (BIPY^2+^ for **M1** and R_1_NHR_2_ for **M2**) could bond identical compound **4** to construct tris[2]pseudorotaxanes through host–guest interaction, respectively; (iii) the host–guest property of metallacycle **M2** with compound **4** could control activate or degenerate by acid or base stimulus. Meanwhile, as an additional benefit, BIPY^2+^ and R_1_NH_2_^+^R_2_ units exhibited different binding ability with compound **4** according to previous literature reports. Thus, acid-activated motion switching of compound **4** between two discrete metallacycles **M1** and **M2** were tried to investigated.

**Figure 5:**
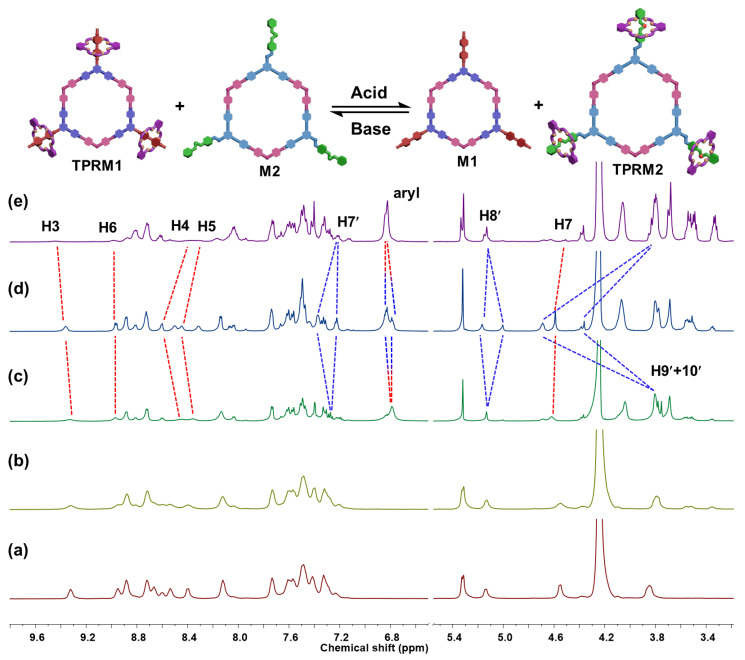
The partial ^1^H NMR spectra (500 MHz, 298 K) in CD_2_Cl_2_/CD_3_NO_2_ (1:1, *v/v*) of the acid-activated motion switching system (**a**) the self-sorting system of metallacycles **M1** and **M2**; (**b**) addition of 0.75 eq DBU to sample a; (**c**) addition of 3.0 eq compound **4** to sample b; (**d**) addition of 3.0 eq TFA to sample c; (**e**) addition of 3.0 eq DBU to sample d.

Firstly, as an acid–base controlled experiments, metallacycle **M2** and compound **4** combined with TFA and DBU (1,8-diazabicyclo[5.4.0]undec-7-ene) to characterize the formation and degradation of the tris[2]pseudorotaxanes **TPRM2**. The ^1^H NMR spectra of the mixtures of metallacycle **M2** and compound **4** with 1:3 ratio in CD_2_Cl_2_/CD_3_NO_2_ (1/1, *v/v*) exhibited that the proton H8’ split into two peaks in which the CD_3_NO_2_ had weak acidity and caused the protonation of R_1_NHR_2_ units to form the small amounts of **TPRM2** (Figure S21b). Subsequently, 0.75 eq. DBU was added to the mixed system to neutralize the acid in the solution, which caused the proton H8’ to return to its original position (Figure S21c). Then TFA was added to this system, giving rise to form tris[2]pseudorotaxanes **TPRM2**. The proton H8’ split into two peaks proved the complex between metallacycle **M2′** and DB24C8. Therefore, the addition of DBU to neutralize the acid in the solution before all studies of molecular motion was quite necessary. Subsequently, acid-activated motion switching of compound **4** between two discrete metallacycles **M1** and **M2** were investigated by ^1^H NMR (Figure 5). When mixing the mixture of metallacycles **M1** and **M2** with the base of DBU in a 1:1:0.75 ratio in CD_2_Cl_2_/CD_3_NO_2_ (1/1, *v/v*), three sets of signals were observed (Figure 5b). After the addition of 3.0 eq. of compound **4** into the mixtures, as indicated in ^1^H NMR spectra (Figure 5c), the proton H4 or H5 on **M1** shifted upfield from 7.150 to 7.192 ppm or 6.987 to 7.013 ppm, respectively, both accompanied with a slight broadening effect, and as a comparison the protons on **M2** remained unchanged, indicating the formation of the self-sorting system containing tris[2]pseudorotaxanes **TPRM1** and metallacycle **M2**. Then, upon adding 3.0 eq. TFA into the system, the protons’ signal on BIPY^2+^ of metallacycle **M1** returned to the initial position and the partial protons’ signal of R_1_NH_2_^+^R_2_ on **M2′** shifted upfield, which was attributed to the stronger binding ability between compound **4** and R_1_NH_2_^+^R_2_ than that of the BIPY^2+^ units (Figure 5d). Thus, a new self-sorting system was established, accompanied by the motion of multiple compound **4** molecules from metallacycle **M1** to metallacycle **M2**. More interestingly, addition of 3.0 eq. DBU to deprotonate of R_1_NH_2_^+^R_2_ units on metallacycle **M2′** to the R_1_NHR_2_, the ^1^H NMR spectrum showed signals corresponding to the compound **4** threaded BIPY^2+^ on the metallacycle **M1** (Figure 5e). Therefore, acid-activated motion switching of compound **4** between two discrete metallacycles **M1** and **M2** was successfully fabricated through host–guest interaction and the self-sorting strategy.

## 3. Materials and Methods

All reagents were commercially available and used as supplied without further purification. Compounds **S1**, **S2**, **S3**, **S4** and **3** were prepared according to the published procedures. Deuterated solvents were purchased from Cambridge Isotope Laboratory (Andover, MA, USA).

All solvents were dried according to standard procedures and all of them were degassed under N_2_ for 30 min before use. All air-sensitive reactions were carried out under inert N_2_ atmosphere. ^1^H NMR, ^13^C NMR and ^31^P NMR spectra were recorded on Bruker 300 MHz Spectrometer (^1^H: 300 MHz; ^31^P: 122 MHz), Bruker 400 MHz Spectrometer (^1^H: 400 MHz; ^13^C: 101 MHz, ^31^P: 162 MHz) and Bruker 500 MHz Spectrometer (^1^H: 500 MHz; ^13^C: 126 MHz, ^31^P: 202 MHz) at 298 K. The ^1^H and ^13^C NMR chemical shifts are reported relative to residual solvent signals, and ^31^P {^1^H} NMR chemical shifts are referenced to an external unlocked sample of 85% H_3_PO_4_ (δ 0.0). 2D NMR spectra (^1^H-^1^H COSY, NOESY and DOSY) were recorded on Bruker 500 MHz Spectrometer (^1^H: 500 MHz) at 298 K. The MALDI MS experiments were carried out on a Bruker UltrafleXtreme MALDI TOF/TOF Mass Spectrometer (Bruker Daltonics, Billerica, MA, USA), equipped with smartbeam-II laser. All spectra were measured in positive reflectron or linear mode.

## 4. Conclusions

In summary, two hexagonal Pt(II) metallacycles **M1** and **M2** decorated with different recognition sites were successfully constructed and exhibited great self-sorting properties. Both of metallacycles **M1** and **M2** could bind identical compound of DB24C8 via host–guest interaction to form different tris[2]pseudorotaxanes **TPRM1** and **TPRM2**, respectively. The constructed tris[2]pseudorotaxanes **TPRM2** systems could suffer from the chemical stimuli of DBU to induce DB24C8 to leave from the binding sites of the metallacycle **M2**, which successfully demonstrated the motion of DB24C8. Based on the host–guest interaction and the self-sorting strategy, the discrete metallacycles **M1** and **M2** have been employed as a unique motion platform for DB24C8 to realize acid-activated motion switching through acid–base stimuli. This study provided a new insight into the design of multiple molecular motion under the influence of chemical stimuli and exhibited potential for constructing stimuli-responsive smart materials.

## Data Availability

Not available.

## Supplementary Materials

The following are available online. Schemes S1–S2: the synthesis of compounds **1** and **2**, Schemes S3–S4: the construction of metallacycles M1 and M2, Figures S1–S6: the 2D NMR spectrum, Figures S7–S18: the characterization of tris[2]pseudorotaxanes TPRM1 and TPRM2, Figures S19–S21: the characterization of acid-activated motion switching, Figures S22–S31: the 1H, 31P, 13C NMR and MS spectra of new compounds. References [61,62,63,64,65] are cited in the supplementary materials.

## Sample Availability

Samples of the compounds are not available from the authors.
