# Supplementary material for: Acid-Activated Motion Switching of DB24C8 between Two Discrete Platinum(II) Metallacycles
Source: Molecules. 2021 Jan 30;26(3):716. doi: 10.3390/molecules26030716 (PMC7866548; doi:10.3390/molecules26030716)
Supplement: Supplementary file 1 [file molecules-26-00716-s001.pdf]

molecules

Supporting Information

Acid-Activated Motion Switching of DB24C8 between two Discrete Platinum(ii) Metallacycles

Yi-Xiong Hu [1,†], Gui-Yuan Wu [1,2,†], Xu-Qing Wang [1], Guang-Qiang Yin [3], Chang-Wei Zhang [1], Xiaopeng Li [3], Lin Xu [1,*] and Hai-Bo Yang [1,*]

[1] Shanghai Key Laboratory of Green Chemistry and Chemical Processes, School of Chemistry and Molecular Engineering, East China Normal University, 3663 N. Zhongshan Road, Shanghai 200062, China; 52184300024@stu.ecnu.edu.cn (Y.-X.H.); wgy@ahnu.edu.cn (G.-Y.W.); xqwang@chem.ecnu.edu.cn (X.-Q.W.); cwzhang@chem.ecnu.edu.cn (C.-W.Z.)
[2] Anhui Province Key Laboratory of Optoelectronic Material Science and Technology, School of Physics and Electronic Information, Anhui Normal University, Wuhu, Anhui 241002, China
[3] College of Chemistry and Environmental Engineering, Shenzhen University, Shenzhen 518055, China; 13162729689@163.com (G.-Q.Y.); xiaopengli@szu.edu.cn (X.L.)
* Correspondence: lxu@chem.ecnu.edu.cn (L.X.); hbyang@chem.ecnu.edu.cn (H.-B.Y.)
† These authors contributed equally to this work.

1. Materials and general methods S2

2. The Synthesis of 120° Donor Precursor 1 and 2 S3

3. The Construction of the Individual Hexagonal Metallacycles M1 and M2 S4

4. The Construction of the Individual Tris[2]pseudorotaxanes S8

5. Acid-activated motion switching S14

6. ^{1}H, ^{31}P, ^{13}C NMR and MS Spectra of New Compounds S16

References S24

1. Materials and General Methods

All reagents were commercially available and used as supplied without further purification, compounds **S1**, **S2**, **S3**, **S4** and **3** were prepared according to the published procedures. Deuterated solvents were purchased from Cambridge Isotope Laboratory (Andover, MA).

All solvents were dried according to standard procedures and all of them were degassed under N_2 for 30 min before use. All air-sensitive reactions were carried out under inert N_2 atmosphere. ^{1}H NMR, ^{13}C NMR and ^{31}P NMR spectra were recorded on Bruker 300 MHz Spectrometer (^{1}H: 300 MHz; ^{31}P: 122 MHz), Bruker 400 MHz Spectrometer (^{1}H: 400 MHz; ^{13}C: 101 MHz, ^{31}P: 162 MHz) and Bruker 500 MHz Spectrometer (^{1}H: 500 MHz; ^{13}C: 126 MHz, ^{31}P: 202 MHz) at 298 K. The ^{1}H and ^{13}C NMR chemical shifts are reported relative to residual solvent signals, and ^{31}P {^{1}H} NMR chemical shifts are referenced to an external unlocked sample of 85% H_3PO_4 (δ 0.0). 2D NMR spectra (^{1}H-^{1}H COSY, NOESY and DOSY) were recorded on Bruker 500 MHz Spectrometer (^{1}H: 500 MHz) at 298 K. The MALDI MS experiments were carried out on a Bruker UltrafleXtreme MALDI TOF/TOF

Mass Spectrometer (Bruker Daltonics, Billerica, MA), equipped with smartbeam-II laser. All spectra were measured in positive reflectron or linear mode.

2. The Synthesis of 120° Donor Precursor 1 and 2

Scheme S1. The Synthesis of 120° Donor Precursor **1**.

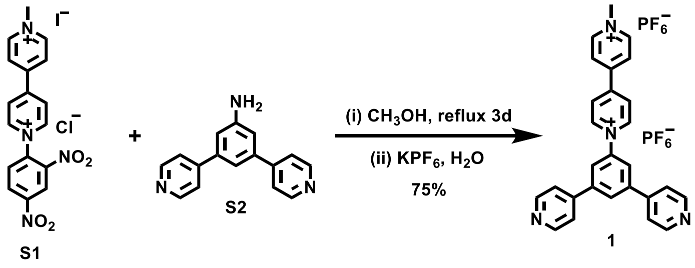

Scheme 1. Compound **S1** [1] (200 mg, 0.40 mmol) was reacted with compound **S2** [2] (395 mg, 1.6 mmol) at reflux for 3 day in 8 mL of methanol. After cooling to room temperature, added 30 mL H_2O to the reaction system, the mixture was filtered and the solvent was removed to give a crude product. Then the residue was dissolved in methanol (5 mL), precipitated by ethyl ether, and the solid was collected by filtration, washed with ethanol and ethyl ether. The collected solid resolved in water and added the saturated aqueous solution of KPF_6 to precipitated the product. The mixture was centrifuged, washed several times with water, and dried. White solid product **1** was obtained by removing the solvent under vacuum. Yield: 207 mg, 75%. ^{1}H NMR (500 MHz, acetone-d_6): δ 9.95-9.94 (d, J = 5 Hz, 2H), 9.45-9.44 (d, J = 5 Hz, 2H), 9.12–9.10 (d, J = 5 Hz, 2H), 8.96–8.95 (d, J = 5 Hz, 2H), 8.78–8.76 (dd, J_1 = 2 Hz, J_2 =4.5 Hz, 4H), 8.65–8.64 (t, J = 1.5 Hz, 1H), 8.59 (d, J = 1.5 Hz, 2H), 7.96–7.95 (dd, J_1 = 1.5 Hz, J_2 = 4.5 Hz, 4H), 4.79 (s, 3H). ^{13}C NMR (126 MHz, acetone-d_6): δ 152.24, 151.63, 150.33, 148.02, 147.35, 146.12, 145.08, 142.35, 129.68, 128.14, 127.97, 124.87, 122.61, 49.54. MALDI-TOF-MS: calcd for [**1** − PF_6^-]$^+$: 547.1; Found: 547.2.

Scheme S2. The Synthesis of 120° Donor Precursor **2**.

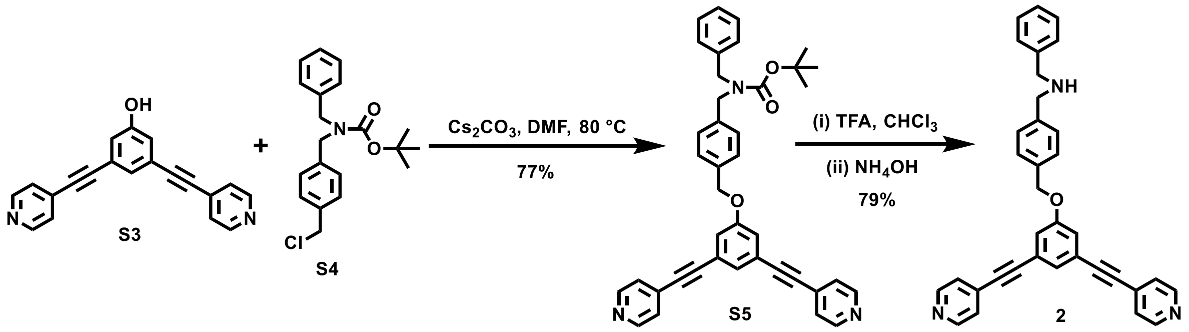

Scheme S5. A 50 mL Schlenk flask was charged with compound **S3** [3] (342 mg, 1.15mmol), Cs_2CO_3 (769 mg, 4.05 mmol), **S4** [4] (200 mg, 0.578 mmol), degassed, and back-filled three times with N_2. Anhydrous DMF (15 mL) were introduced into the reaction flask by syringe. The reaction was stirred under an inert atmosphere at 80 °C for a night. The solvent was taken up in CH_2Cl_2/H_2O mixture (100/50 mL). The organic phase was washed with H_2O (3 × 100 mL). The organic phases were collected and dried over Na_2SO_4, and the solution was evaporated in vacuum. After column chromatography on SiO_2 (CH_2Cl_2/CH_3OH), compound **S5** was obtained in 77% yield (270 mg). ^{1}H NMR (500 MHz, acetone-d_6): δ 8.66–8.65 (dd, J_1 = 2 Hz, J_2 = 4.5 Hz, 4H), 7.51–7.50 (m, 6H), 7.43 (t, J = 1.5 Hz, 1H), 7.42–7.27 (m, 9H), 5.26 (s, 2H), 4.46 (s, 2H), 4.41 (s, 4H), 1.47 (s, 9H). ^{13}C NMR (126 MHz, acetone-d_6): δ 159.87, 156.37, 150.98, 139.39, 136.50, 131.23, 129.32, 128.76, 128.41, 127.99, 126.22, 124.67, 120.00, 92.77, 88.04, 80.20, 70.77, 50.43, 50.10, 49.78, 28.55. ESI-TOF-MS: calcd for [**S5**]$^+$: 605.74; Found: 605.81.

Synthesis of 2: To a solution of compound **S5** (500 mg, 0.825 mmol) in $CHCl_3$ was added TFA (2.5 mL). The mixture was stirred at room temperature for 3 h, and then NH_4OH was added dropwise until the solution was at pH 7. The mixture was extracted with CH_2Cl_2 (50 mL), the organic layer was washed with water (3 × 100 mL). Collected the organic layer and dried over anhydrous sodium sulfate, and the solvent was evaporated in vacuum. Compound **2** was obtained in 79% yield (370 mg). ^{1}H NMR (500 MHz, acetone-d_6): δ 8.66–8.65 (dd, J_1 = 1.5 Hz, J_2 = 4.5 Hz, 4H), 7.51–7.44 (m, 8H), 7.42–7.39 (m, 3H), 7.35–

7.30 (m, 4H), 7.25–7.22 (t, J = 2 Hz, 1H), 5.25 (s, 2H), 3.81 (s, 2H), 3.80 (s, 4H). ^{13}C NMR (126 MHz, acetone-d_6): δ 159.92, 150.98, 142.06, 141.95, 135.88, 131.23, 129.11, 129.01, 128.90, 128.54, 128.36, 127.45, 126.22, 124.65, 119.99, 92.78, 88.02, 70.91, 53.64, 53.33. ESI-TOF-MS: calcd for [**2**]$^+$: 505.62; Found: 505.83.

3. The Construction of the Individual Hexagonal Metallacycles M1 and M2

Scheme S3. The Synthesis of Metallacycles **M1**.

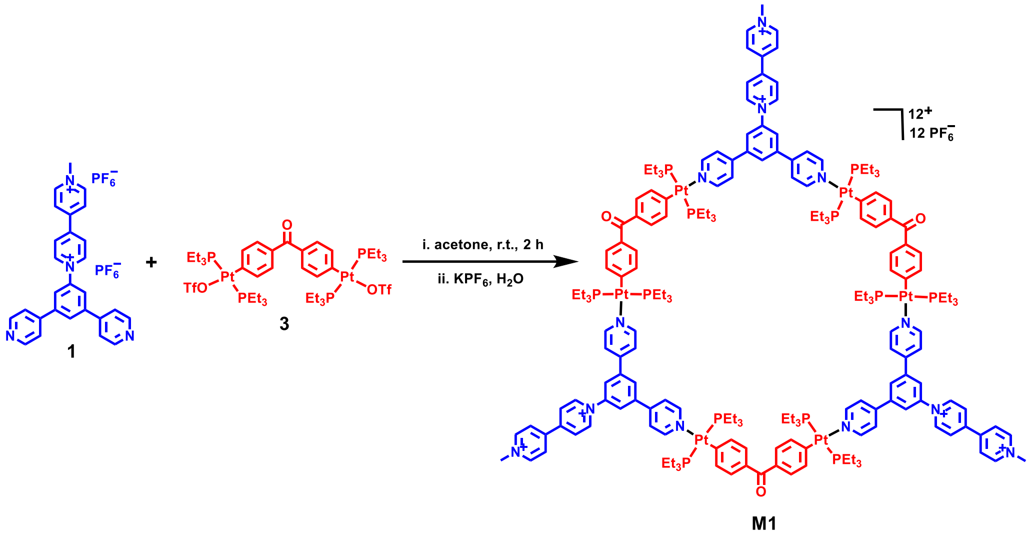

Synthesis of M1: The dipyridyl donor ligand **1** (7.30 mg, 14.44 μmol) and the 120° diplatinum acceptor **3** [5] (20.39 mg, 16.78 μmol) were weighed accurately into a glass vial. To the vial was added 1.0 mL of acetone, and the reaction solution was then stirred at room temperature for 2 h to yield a homogeneous solution. Then the addition of a saturated aqueous solution of KPF_6 into the bottle with continuous stirring (10 min) precipitated the product. The reaction mixture was centrifuged, washed several times with water, and dried. **M1** was obtained by removing the solvent under vacuum. ^{1}H NMR (500 MHz, acetone-d_6): δ 9.81-9.80 (d, J = 5 Hz, 6H), 9.46–9.45 (d, J = 5 Hz, 6H), 9.16–9.15 (d, J = 5 Hz, 12H), 9.06–9.05 (d, J = 5 Hz, 6H), 8.95–8.93 (d, J = 10 Hz, 6H), 8.91(s, 3H), 8.79 (s, 6H), 8.40–8.39 (d, J = 5 Hz, 12H), 7.75–7.73 (d, J = 5 Hz, 12H), 7.61–7.59 (d, J = 5 Hz, 12H), 4.81 (s, 9H), 1.54–1.52 (m, 72H), 1.25–1.18 (m, 108H). ^{13}C NMR (126 MHz, acetone-d_6): δ 196.07, 153.93, 152.49, 150.33, 149.08, 148.03, 147.27, 145.25, 143.03, 140.42, 137.07, 134.19, 130.57, 130.09, 128.18, 128.00, 126.71, 100.88, 49.55, 13.31, 13.18, 13.04, 7.86. ^{31}P NMR (202 MHz, acetone-d_6): δ 14.20 (s, $^1J_{Pt\text{-}P}$ = 2656.3 Hz). MS (ESI-MS): m/z calcd for $[M - 4PF_6^-]^{4+}$: 1374.0639, found: 1373.9945; m/z calcd for $[M - 5PF_6^-]^{5+}$: 1070.2583, found: 1070.2709.

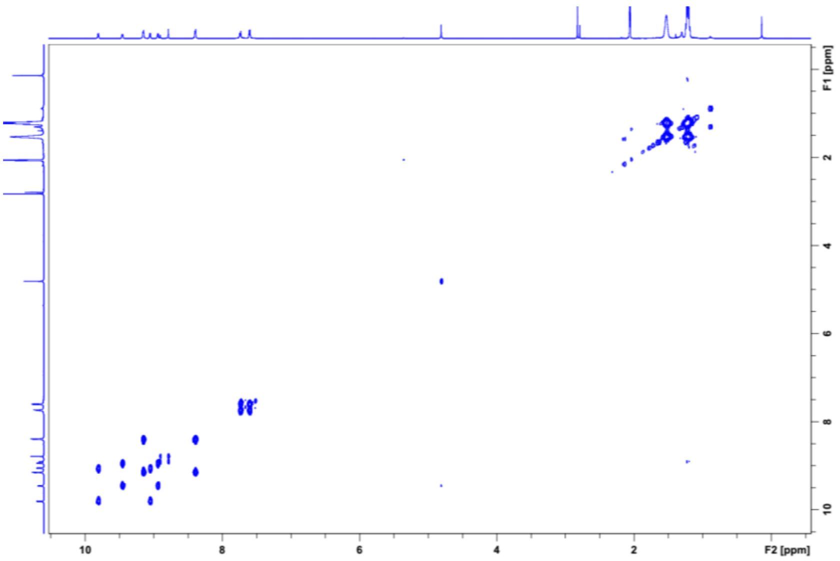

Figure S1. The 2D COSY NMR (500 MHz, acetone-*d*6, 298 K) spectrum of metallacycle **M1**.

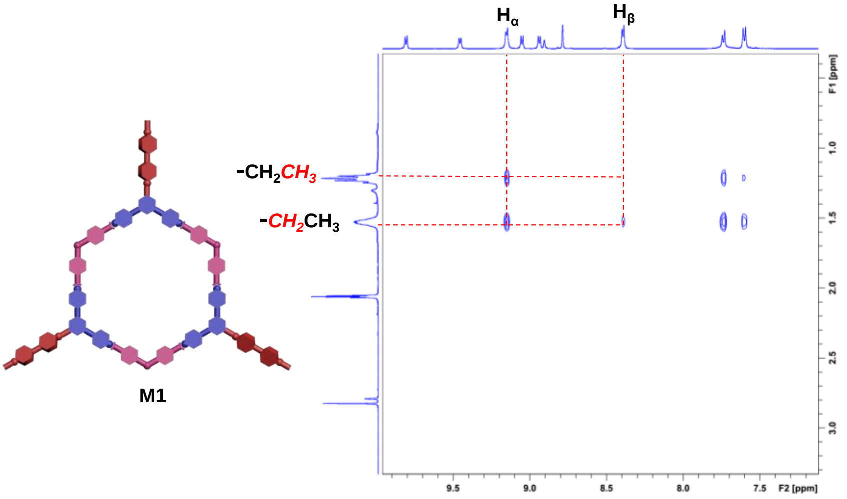

Figure S2. The partial 2D NOESY NMR (500 MHz, acetone-*d*6, 298 K,) spectrum of metallacycle **M1** (The signals in spectra indicated the formation of Pt-N bonds).

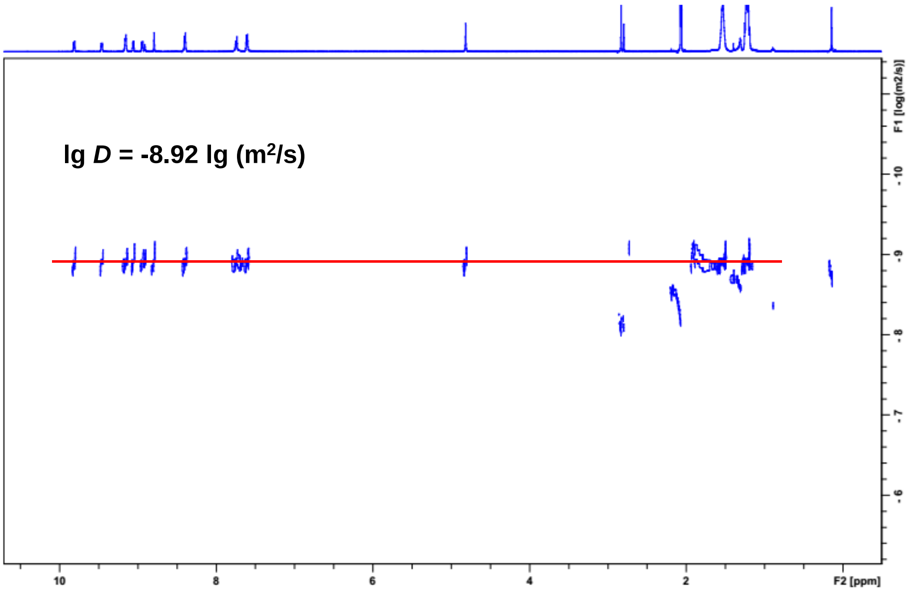

Figure S3. 2D DOSY NMR (500 MHz, acetone-*d*6, 298 K) spectrum of metallacycle **M1**.

Scheme S4. The Synthesis of Metallacycles **M2**.

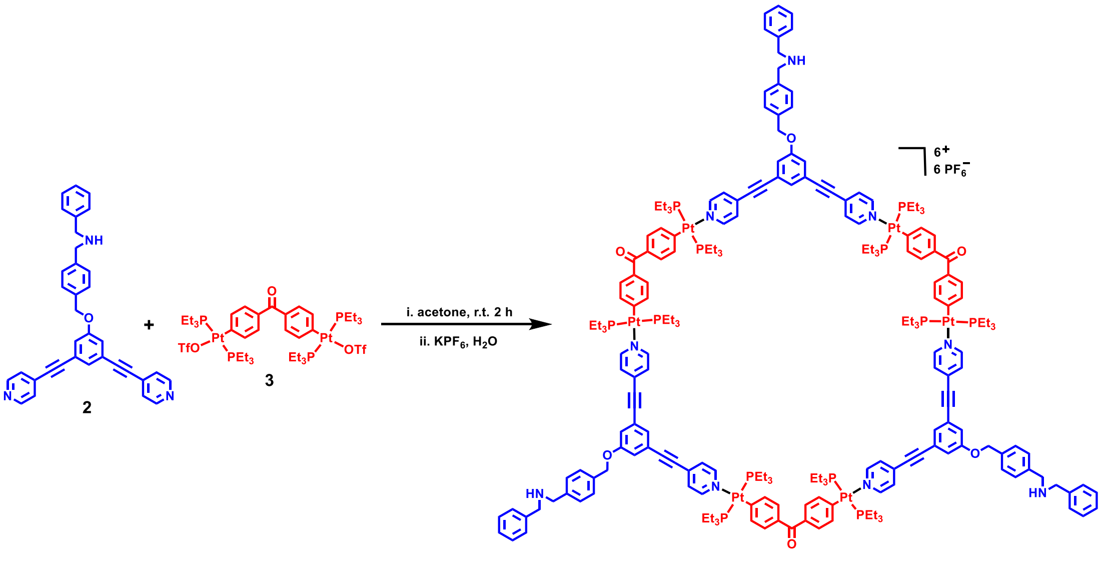

Synthesis of M2: The dipyridyl donor ligand **2** (7.30 mg, 14.44 μmol) and the 120° diplatinum acceptor **3** (19.37 mg, 14.44 μmol) were weighed accurately into a glass vial. were weighed accurately into a glass vial. To the vial was added 1.0 mL of acetone, and the reaction solution was then stirred at room temperature for 2 h to yield a homogeneous solution. Then the addition of a saturated aqueous solution of KPF_6 into the bottle with continuous stirring (10 min) precipitated the product. The reaction mixture was centrifuged, washed several times with water, and dried. **M2** was obtained by removing the solvent under vacuum. ^{1}H NMR (400 MHz, acetone-d_6): δ 9.10–9.09 (d, J = 4 Hz, 12H), 7.95–7.93 (d, J = 8 Hz, 12H), 7.72–7.70 (d, J = 8 Hz, 12H), 7.60–7.56 (m, 15H), 7.49 (s, 18H), 7.41–7.40 (d, J = 4 Hz, 6H), 7.35–7.31 (t, J = 8 Hz, 6H), 7.27–7.23 (t, J = 8 Hz, 3H), 5.28 (s, 6H), 3.85 (s, 6H), 3.82 (s, 6H), 1.53–1.51 (m, 72H), 1.25–1.17 (m, 108H). ^{13}C NMR (126 MHz, acetone-d_6): δ 196.03, 160.06, 153.52, 142.82, 142.75, 142.67, 142.02, 141.74, 137.01, 135.73, 134.74, 134.20, 130.28, 130.10, 129.21, 129.05, 128.94, 128.62, 127.55, 124.04, 121.05, 116.08, 115.89, 97.03, 86.99, 71.07, 53.62, 53.28, 13.27, 13.14, 13.00, 7.87. ^{31}P NMR (202 MHz, acetone-d_6): δ 14.54 (s, $^1J_{Pt\text{-}P}$ = 2650.24 Hz). MS (ESI-MS): m/z calcd for $[M - 4PF_6^-]^{4+}$: 1233.9576, found: 1233.9489; m/z calcd for $[M - 5PF_6^-]^{5+}$: 958.1732, found: 958.2781.

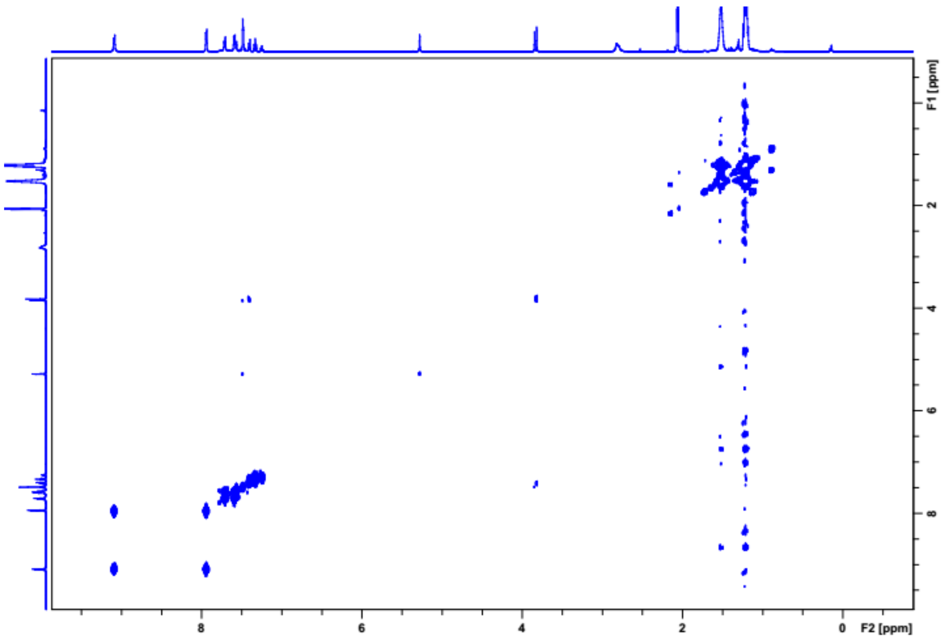

Figure S4. The 2D COSY NMR (500 MHz, acetone-d_6, 298 K) spectrum of metallacycle **M2**.

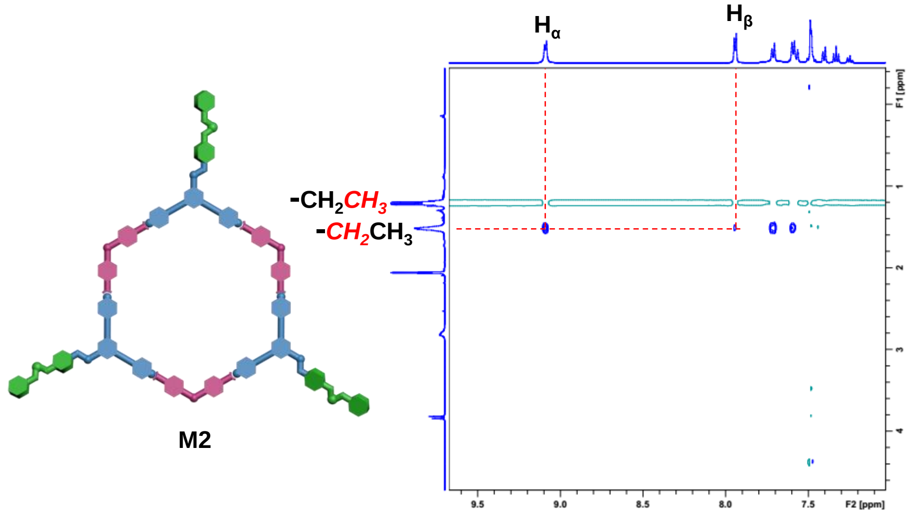

Figure S5. The partial 2D NOESY NMR (500 MHz, acetone-d_6, 298 K,) spectrum of metallacycle **M2** (The signals in spectra indicated the formation of Pt-N bonds).

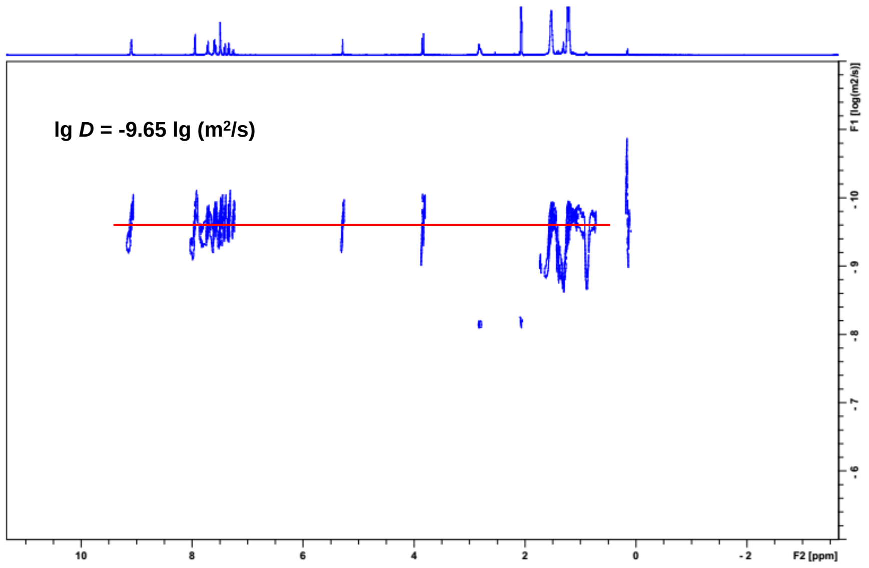

Figure S6. 2D DOSY NMR (500 MHz, acetone-d_6, 298 K) spectrum of metallacycle **M2**

4. The Construction of the Individual Tris[2]pseudorotaxanes

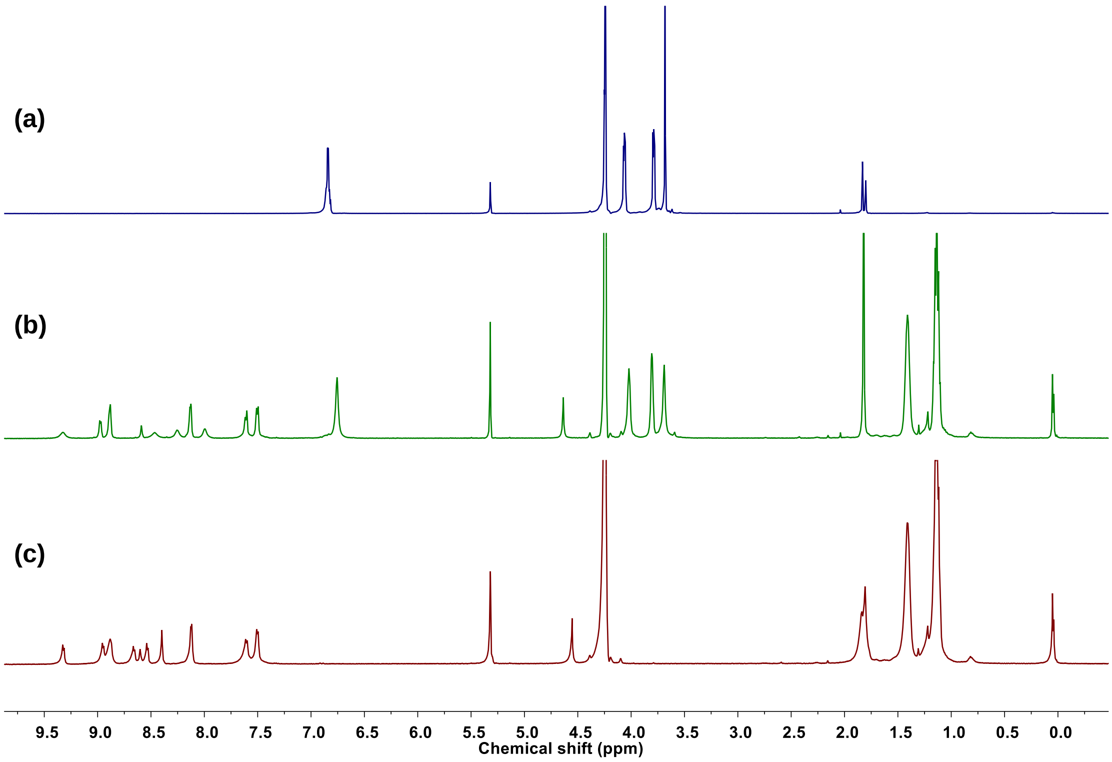

Figure S7. The ^{1}H NMR spectra (500 MHz, 298 K) of compound **4** (**a**), tris[2]pseudorotaxanes **TPRM1** (**b**) and metallacycle **M1** (**c**) in CD_2Cl_2/CD_3NO_2 (1/1, *v*/*v*).

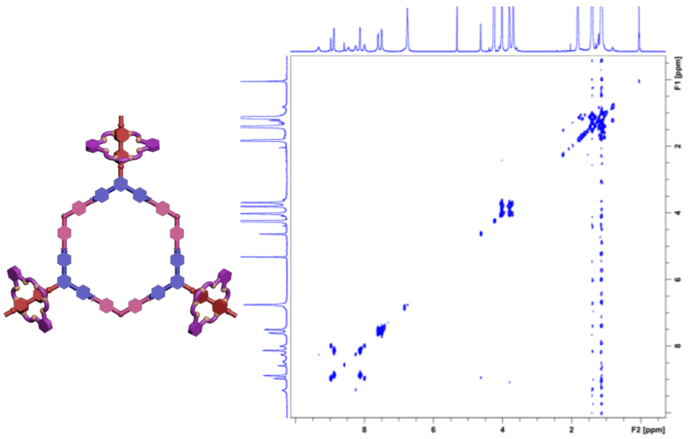

Figure S8. The 2D COSY NMR (500 MHz, CD_2Cl_2/CD_3NO_2 (1/1, *v*/*v*), 298 K) spectrum of the tris[2]pseudorotaxanes **TPRM1**.

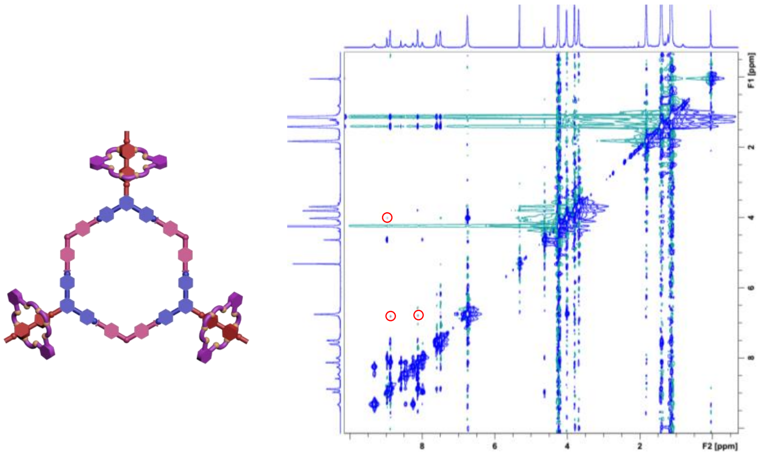

Figure S9. The 2D NOESY NMR (500 MHz, CD_2Cl_2/CD_3NO_2 (1/1, *v*/*v*), 298 K) spectrum of the tris[2]pseudorotaxanes **TPRM1**.

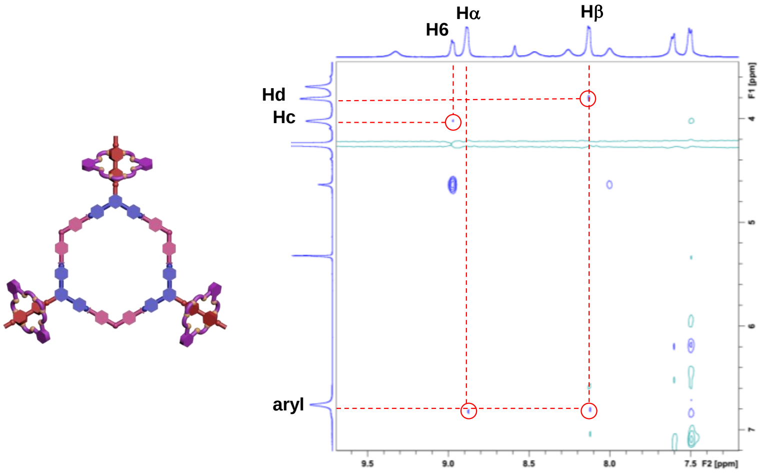

Figure S10. The partial 2D NOESY NMR (500 MHz, CD_2Cl_2/CD_3NO_2 (1/1, *v*/*v*), 298 K) spectrum of the tris[2]pseudorotaxanes **TPRM1**.

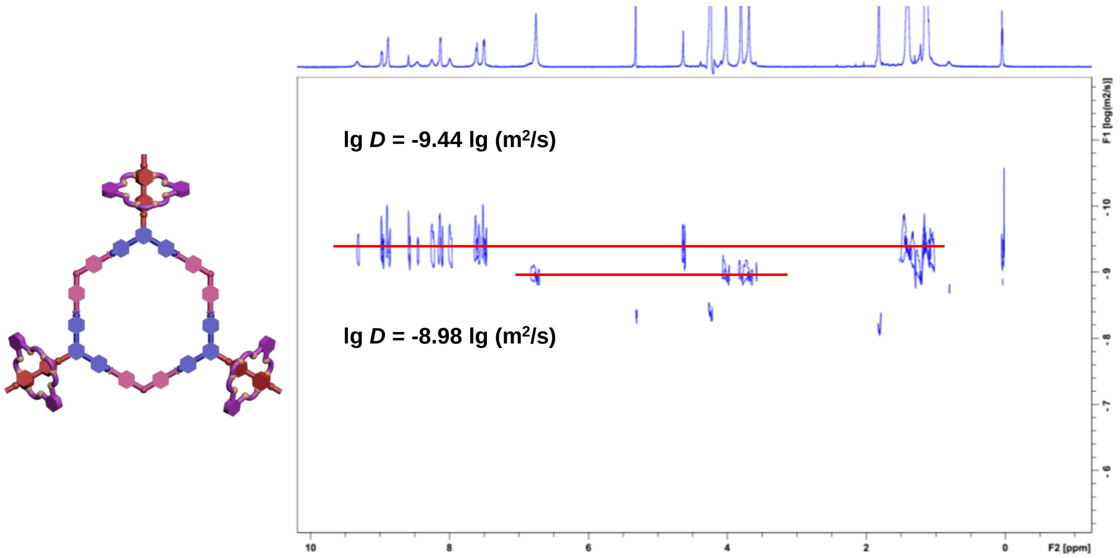

Figure S11. 2D DOSY NMR (500 MHz, CD_2Cl_2/CD_3NO_2 (1/1, *v*/*v*), 298 K) spectrum of the tris[2]pseudorotaxanes **TPRM1**.

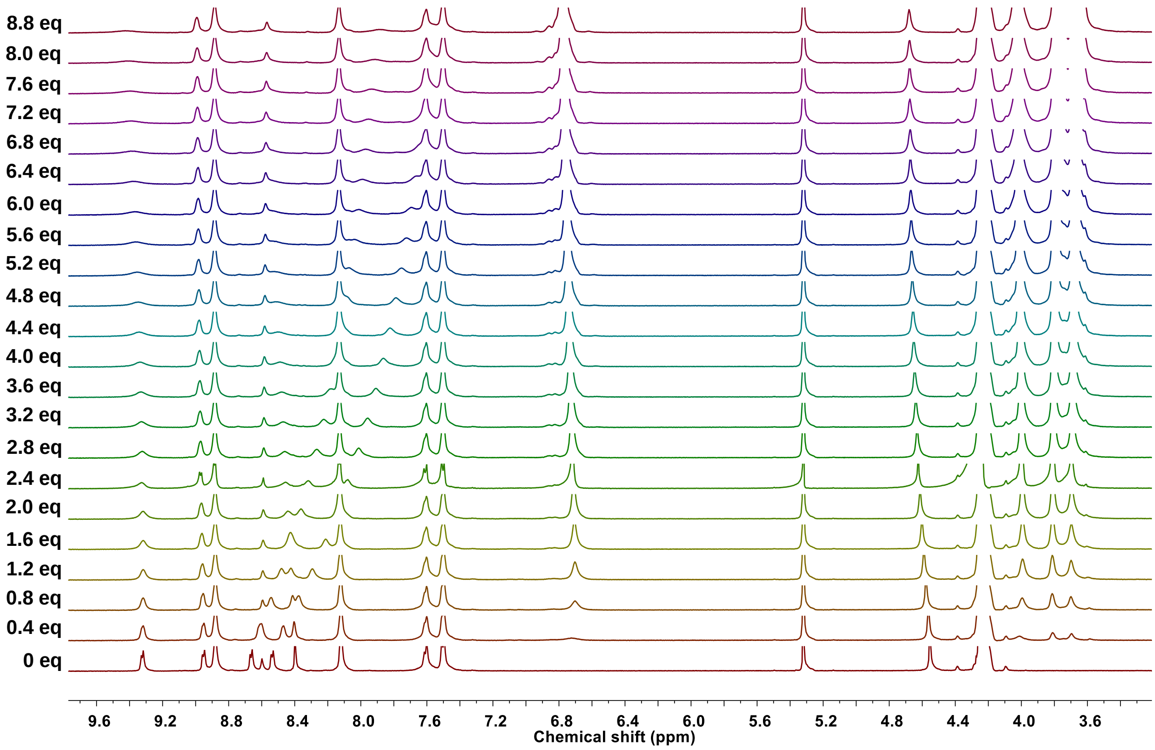

Figure S12. Partial ^1H NMR (500 MHz, CD_2Cl_2/CD_3NO_2 (1/1, *v*/*v*), 298 K) spectrum of the metallacycle **M1** (1.0×10^{-3} M) with additions of compound **4**.

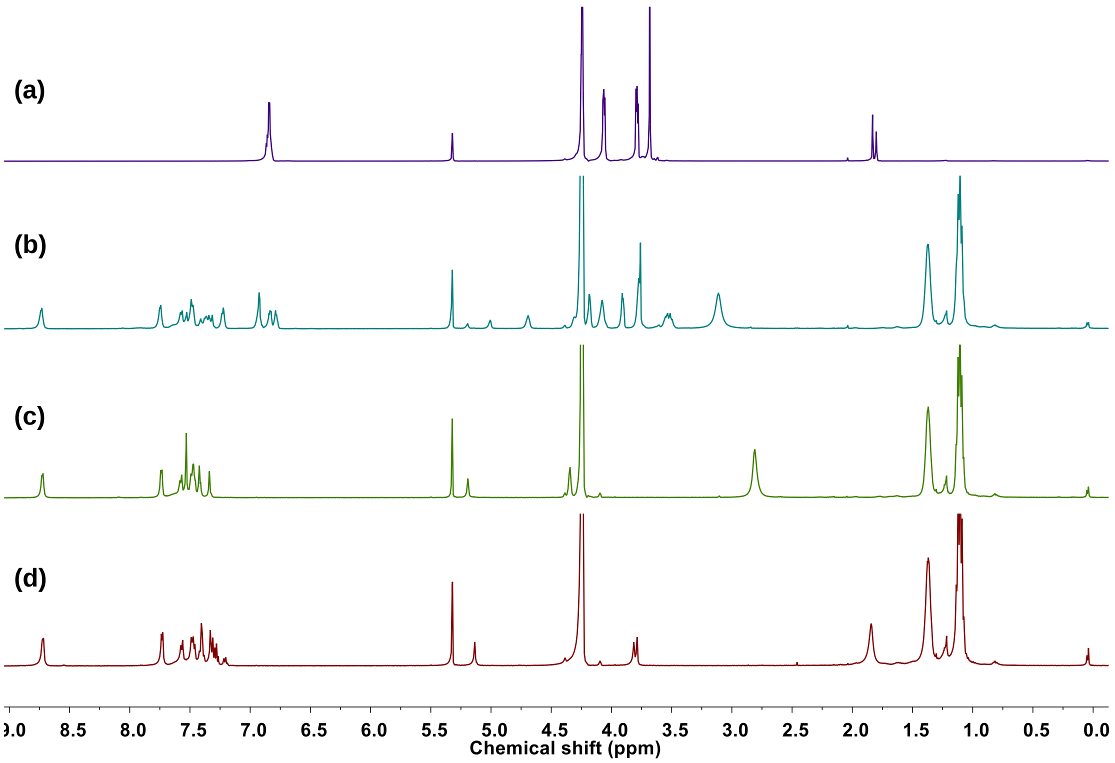

Figure S13. The ^{1}H NMR spectra (500 MHz, 298 K) of compound **4** (**a**), tris[2]pseudorotaxanes **TPRM2** (**b**), metallacycle **M2′** (**c**) and metallacycle **M2** (**d**) in CD_2Cl_2/CD_3NO_2 (1/1, *v*/*v*).

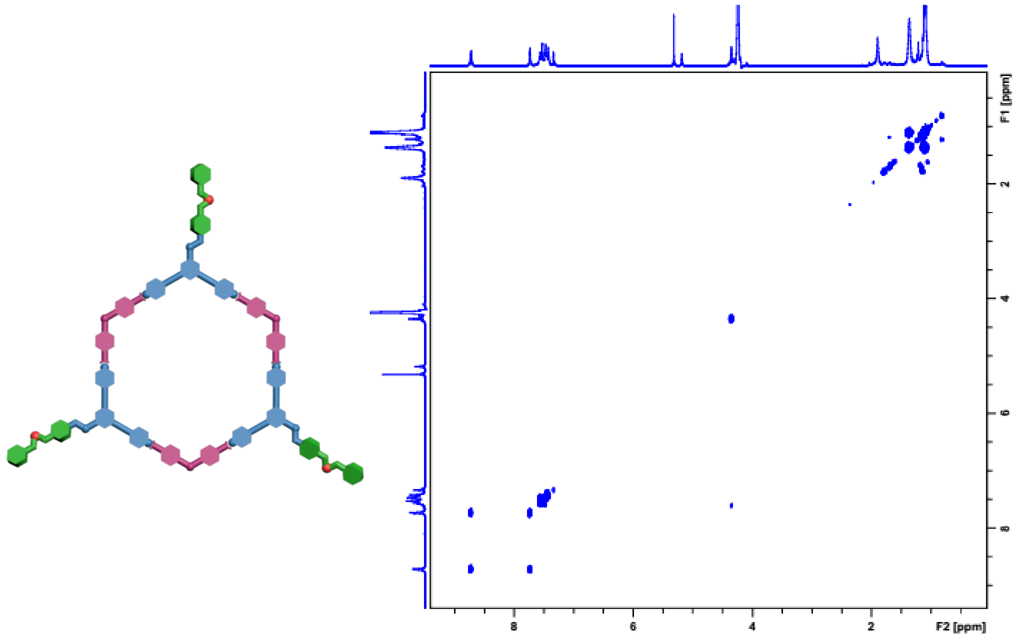

Figure S14. The 2D COSY NMR (500 MHz, CD_2Cl_2/CD_3NO_2 (1/1, *v*/*v*), 298 K) spectrum of the metallacycle **M2′**.

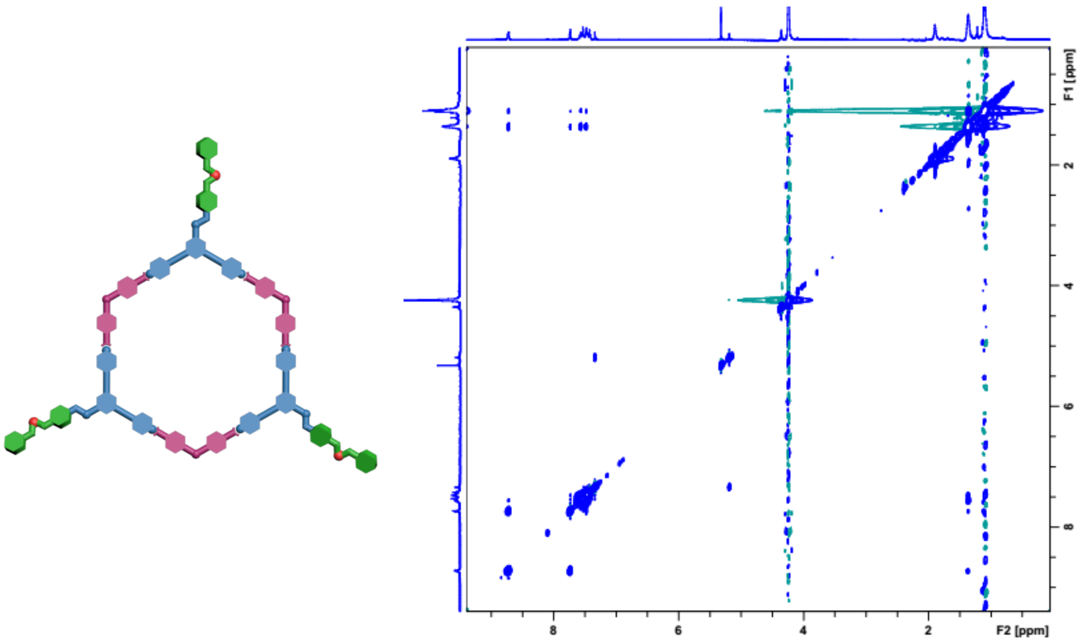

Figure S15. The partial 2D NOESY NMR (500 MHz, CD_2Cl_2/CD_3NO_2 (1/1, *v*/*v*), 298 K) spectrum of the metallacycle **M2′**.

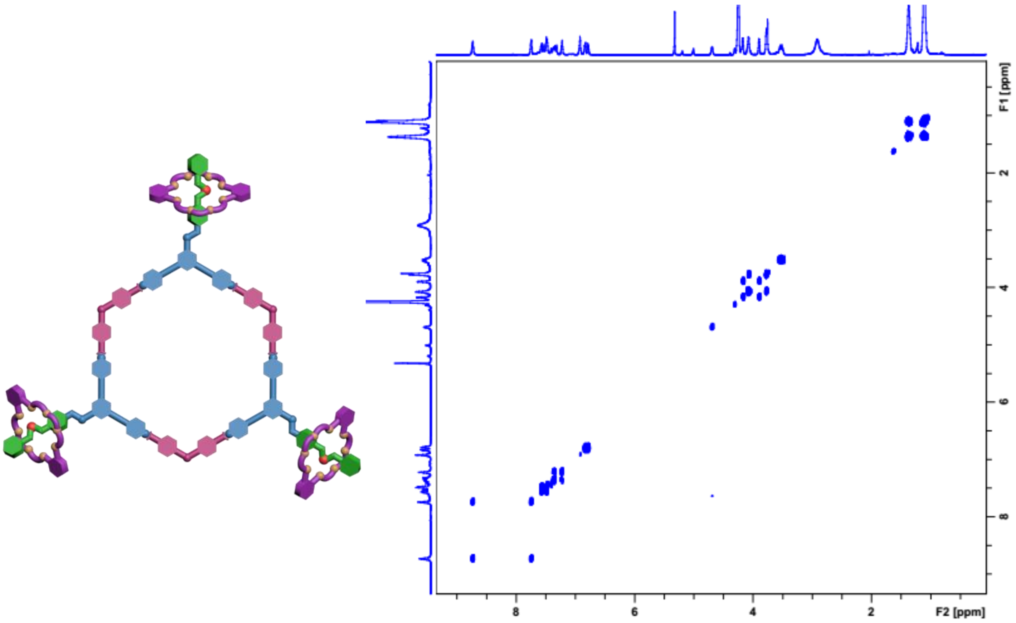

Figure S16. The 2D COSY NMR (500 MHz, CD_2Cl_2/CD_3NO_2 (1/1, *v*/*v*), 298 K) spectrum of the tris[2]pseudorotaxanes **TPRM2**.

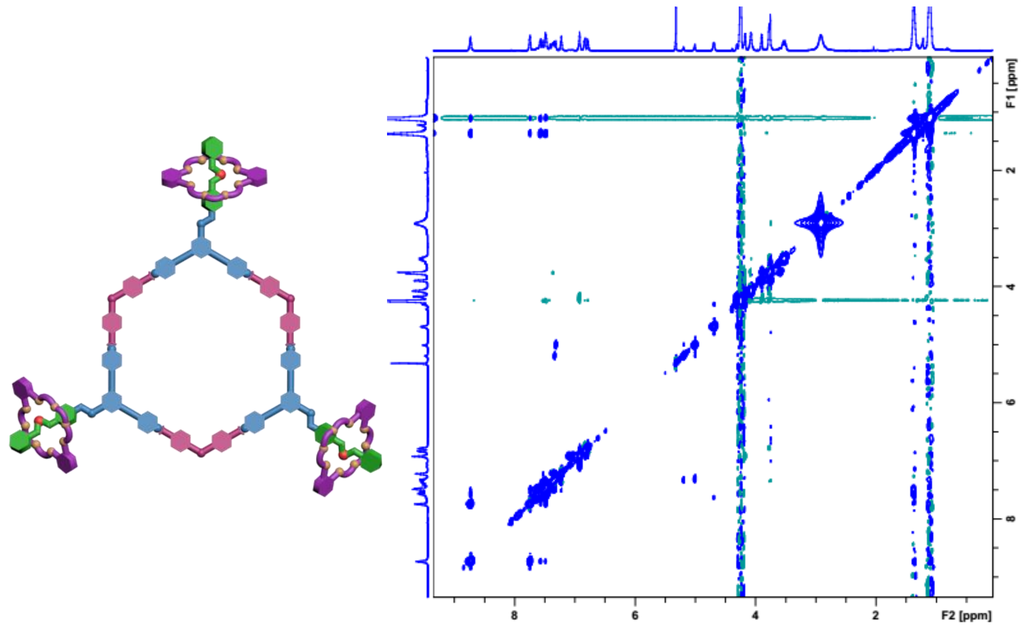

Figure S17. The 2D NOESY NMR (500 MHz, CD_2Cl_2/CD_3NO_2 (1/1, *v*/*v*), 298 K) spectrum of the tris[2]pseudorotaxanes **TPRM2**.

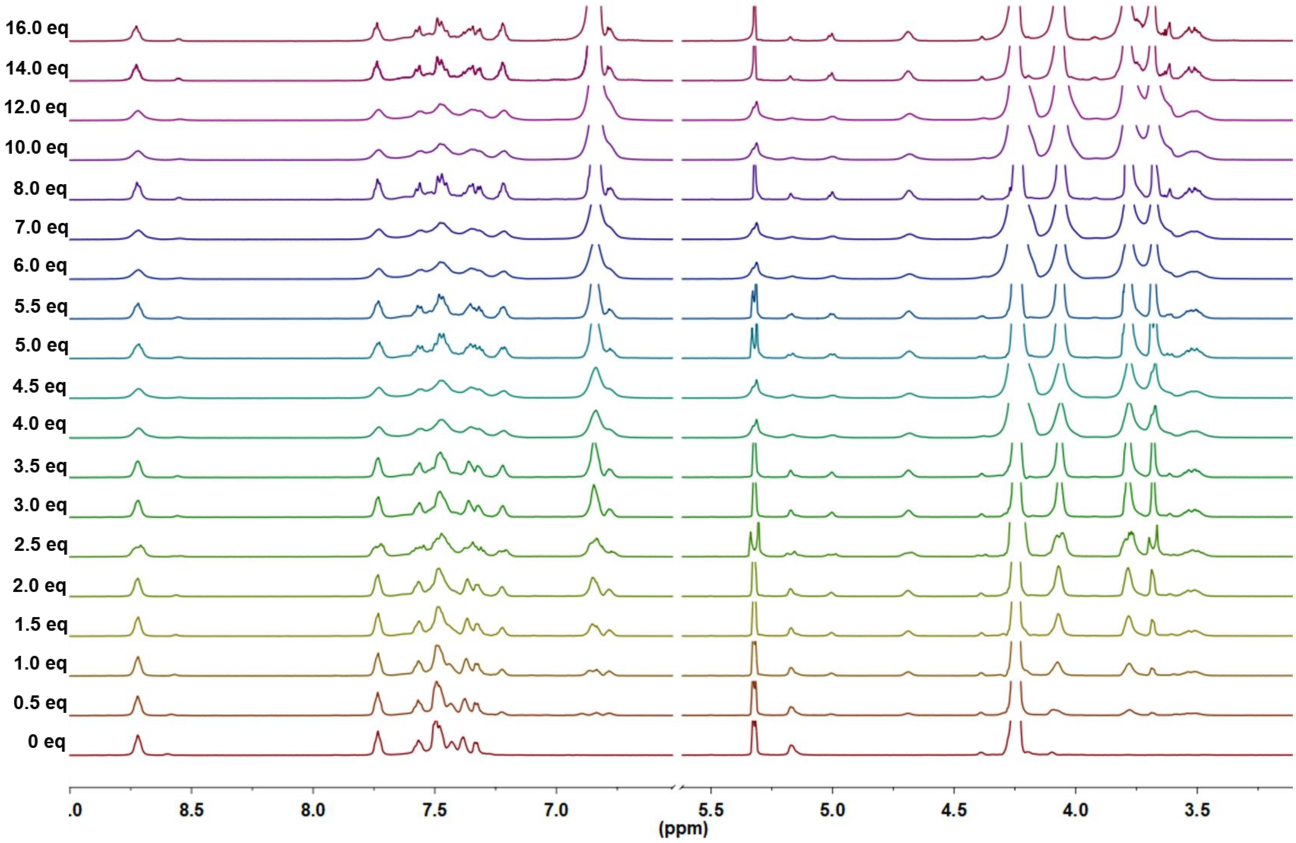

Figure S18. Partial ^{1}H NMR (500 MHz, CD_2Cl_2/CD_3NO_2 (1/1, *v*/*v*), 298 K) spectrum of the metallacycle **M2′** (1.0 × 10^{-3} M) with additions of compound **4**.

5. Acid-Activated Motion Switching

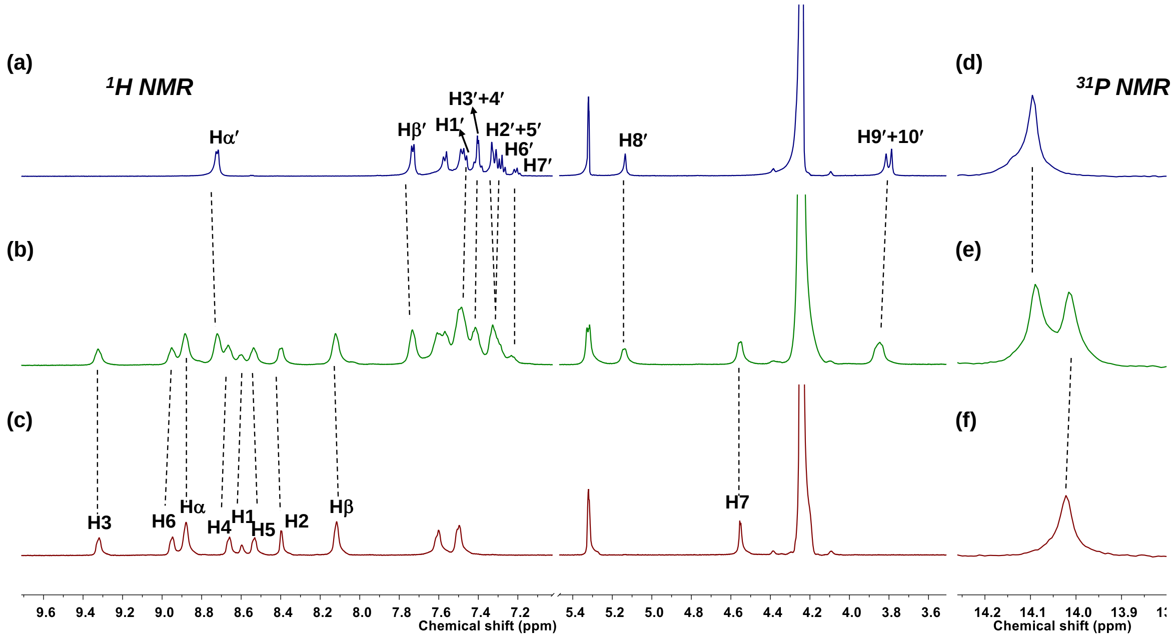

Figure S19. The partial ¹H NMR spectra (500 MHz, in CD_2Cl_2/CD_3NO_2 (1/1, *v*/*v*), 298 K) (*left*) spectra of the individual metallacycle **M2** (**a**), the size-controlled self-sorting system (**b**), and the individual metallacycle **M1** (**c**). The partial ³¹P NMR (202 MHz, 298K) (*right*) spectra of the individual metallacycle **M2** (**d**), the size-controlled self-sorting system (**e**), and the individual metallacycle **M1** (**f**).

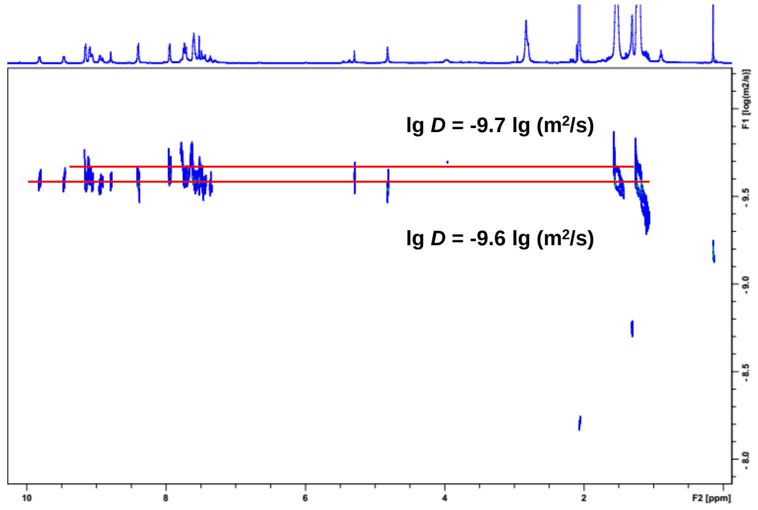

Figure S20. 2D DOSY NMR (500 MHz, acetone-d_6, 298 K) spectrum of the self-sorting system of metallacycles **M1** and **M2**.

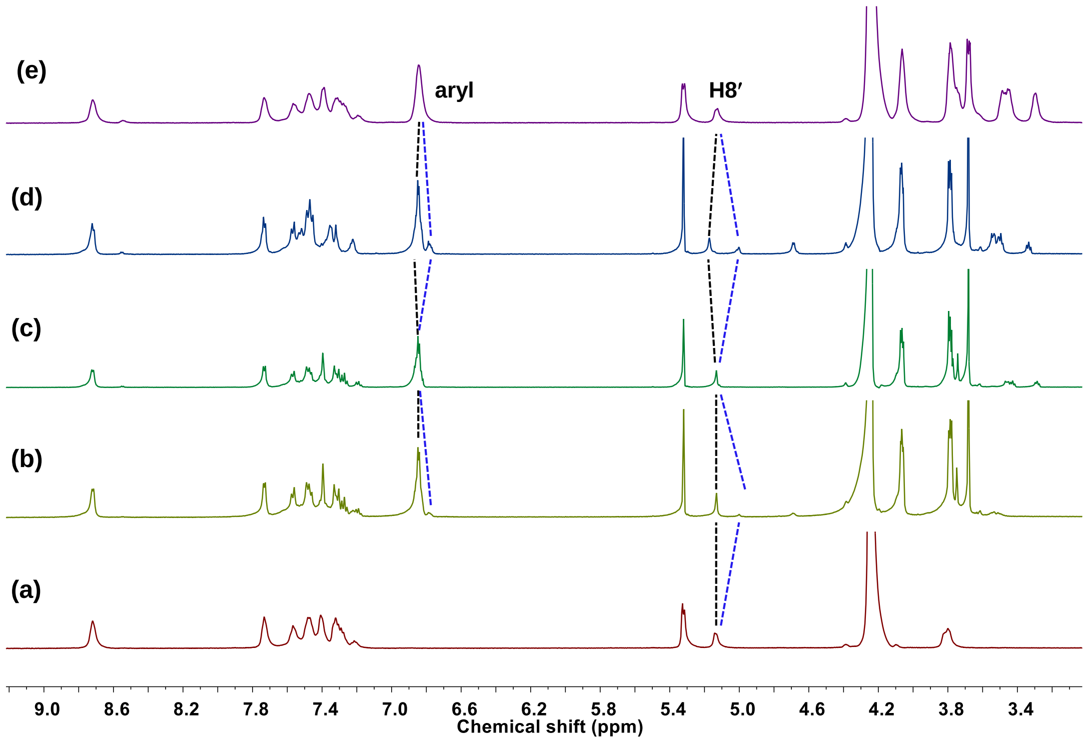

Figure S21. The partial ^{1}H NMR spectra (500 MHz, CD_2Cl_2/CD_3NO_2 (1/1, *v*/*v*), 298 K) of (**a**) the individual metallacycle **M2**. (**b**) addition of 3.0 eq. compound **4** to sample a. (**c**) addition of 0.75 eq. DBU to sample b. (**d**) addition of 3.0 eq. TFA to sample c. (**e**) addition of 3.0 eq. DBU to sample d.

6. ^{1}H, ^{31}P, ^{13}C NMR and MS Spectra of New Compounds

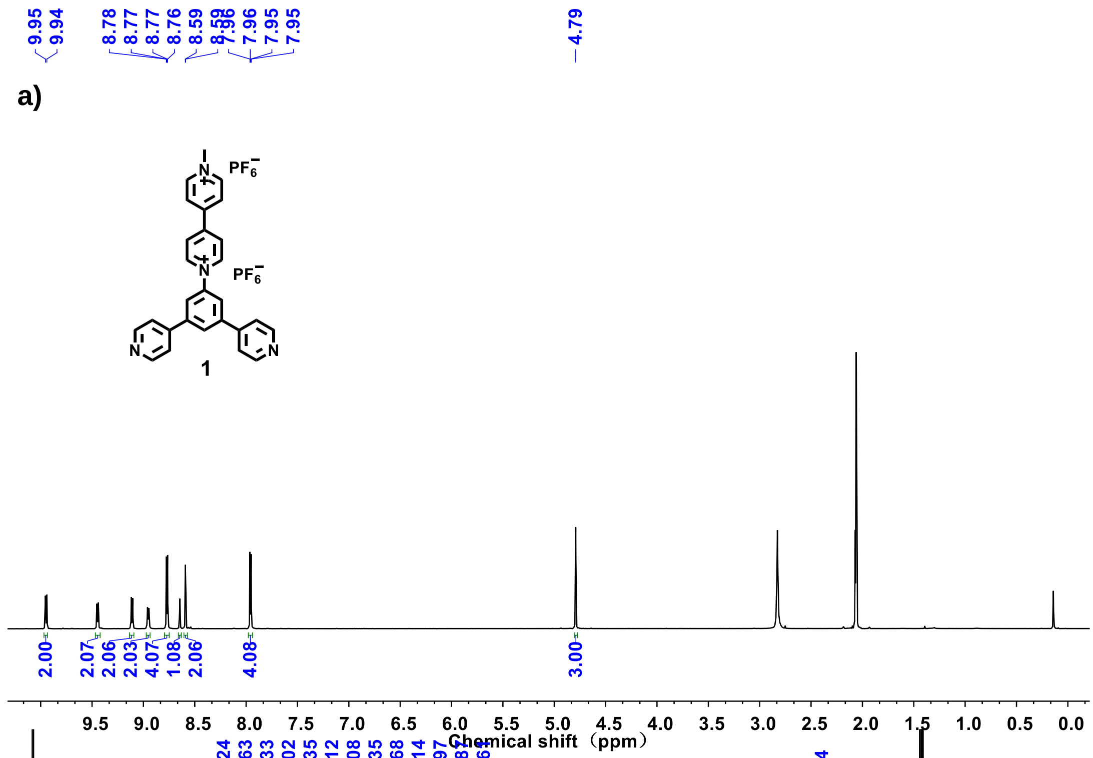

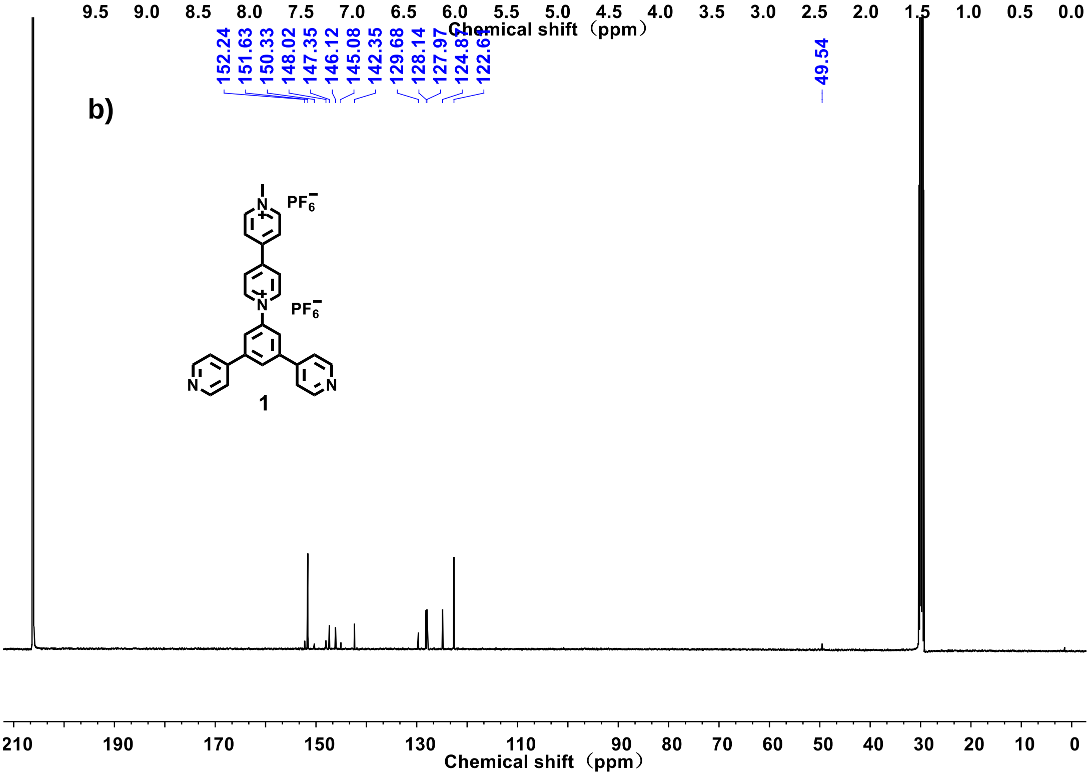

Figure S22. (**a**) ^{1}H NMR spectrum (500 MHz, *d*-acetone, 298 K), (**b**) ^{13}C NMR spectrum (126 MHz, *d*-acetone, 298 K) of compound **1**.

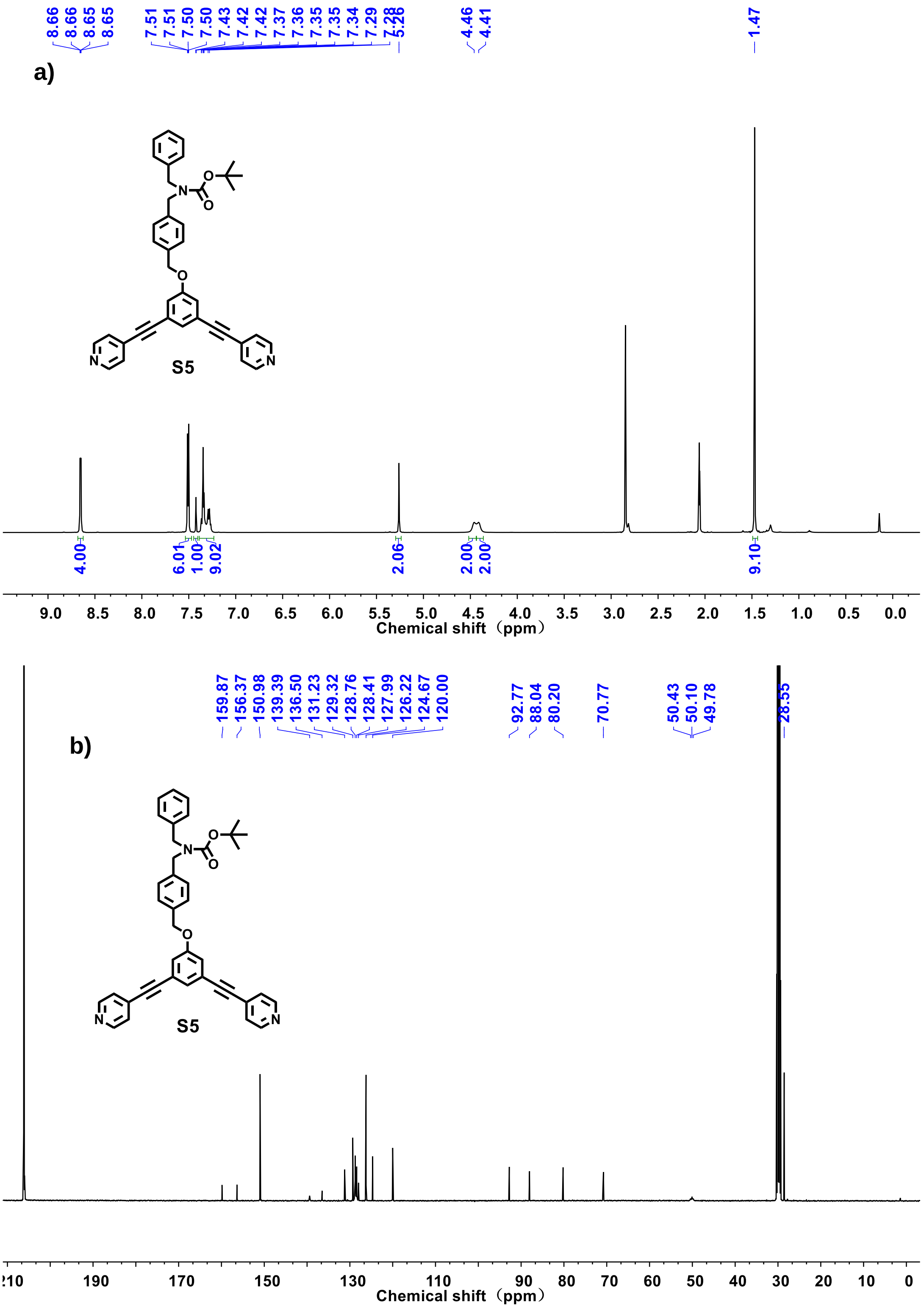

Figure S23. (**a**) ^{1}H NMR spectrum (500 MHz, *d*-acetone, 298 K), (**b**) ^{13}C NMR spectrum (126 MHz, *d*-acetone, 298 K) of compound **S5**.

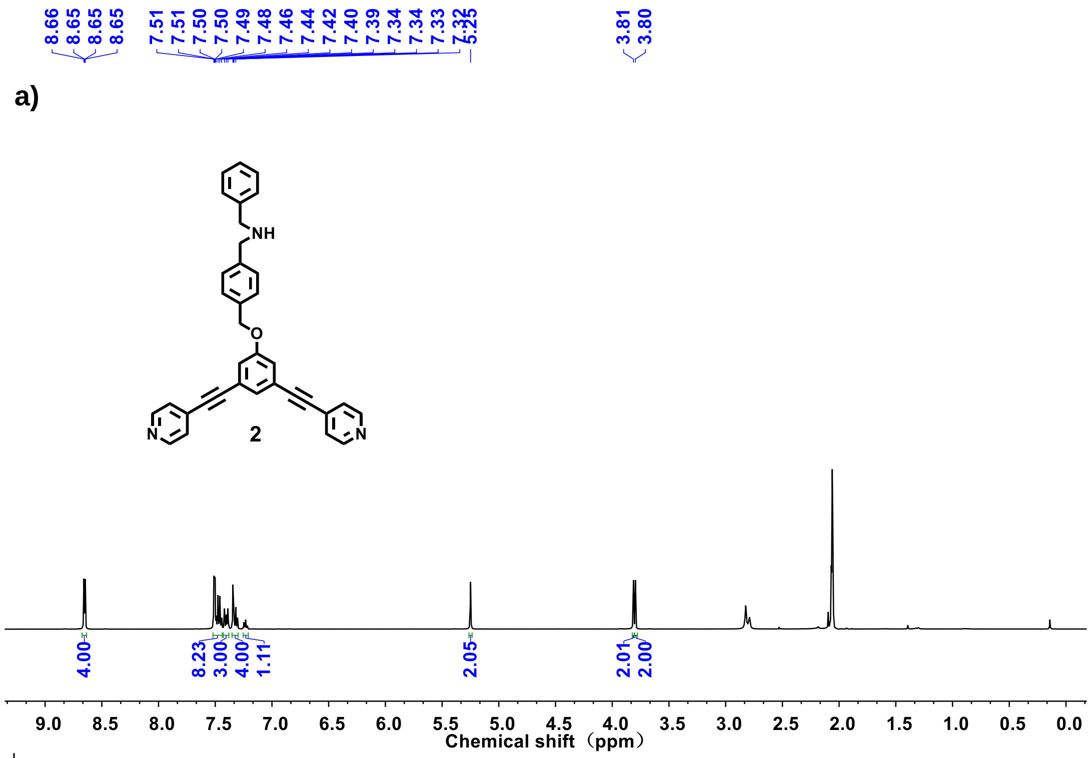

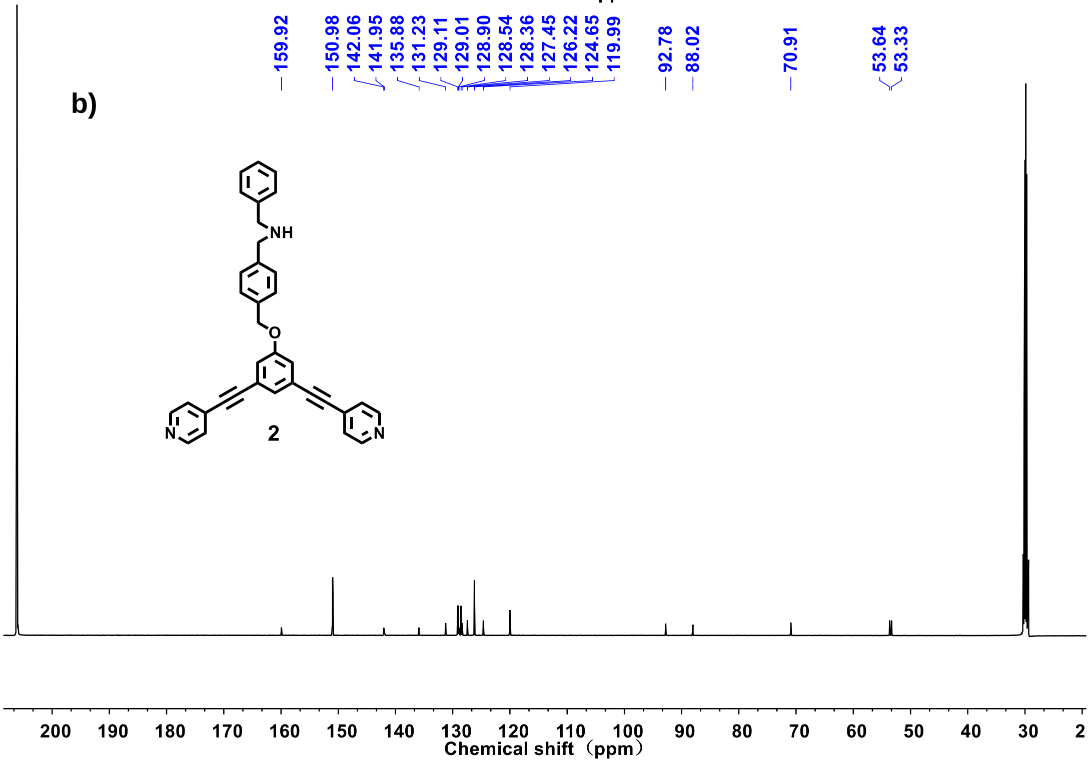

Figure S24. **a**) ^{1}H NMR spectrum (500 MHz, *d*-acetone, 298 K), **b**) ^{13}C NMR spectrum (126 MHz, *d*-acetone, 298 K) of compound **2**.

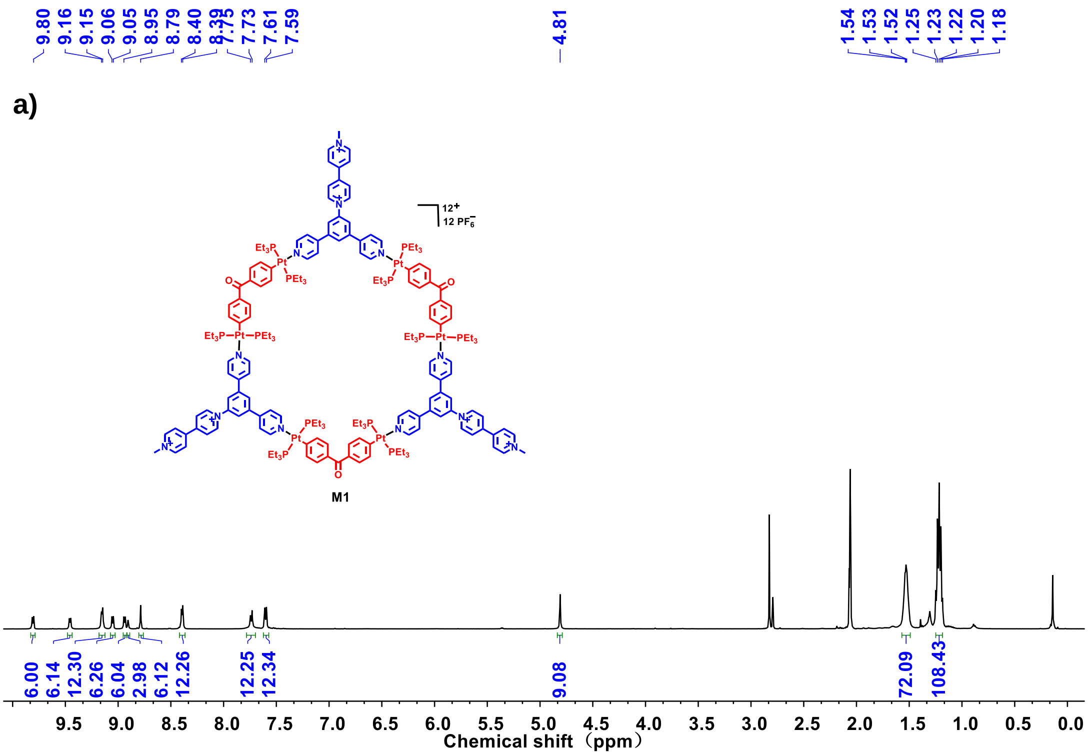
9.80
9.16
9.15
9.06
9.05
8.95
8.79
8.40
8.39
7.75
7.73
7.61
7.59
4.81
1.54
1.53
1.52
1.25
1.23
1.22
1.20
1.18
a)
12+
12 PF6−
M1
6.00
6.14
12.30
6.26
6.04
2.98
6.12
12.26
12.25
12.34
9.08
72.09
108.43
9.5 9.0 8.5 8.0 7.5 7.0 6.5 6.0 5.5 5.0 4.5 4.0 3.5 3.0 2.5 2.0 1.5 1.0 0.5 0.0
Chemical shift (ppm)

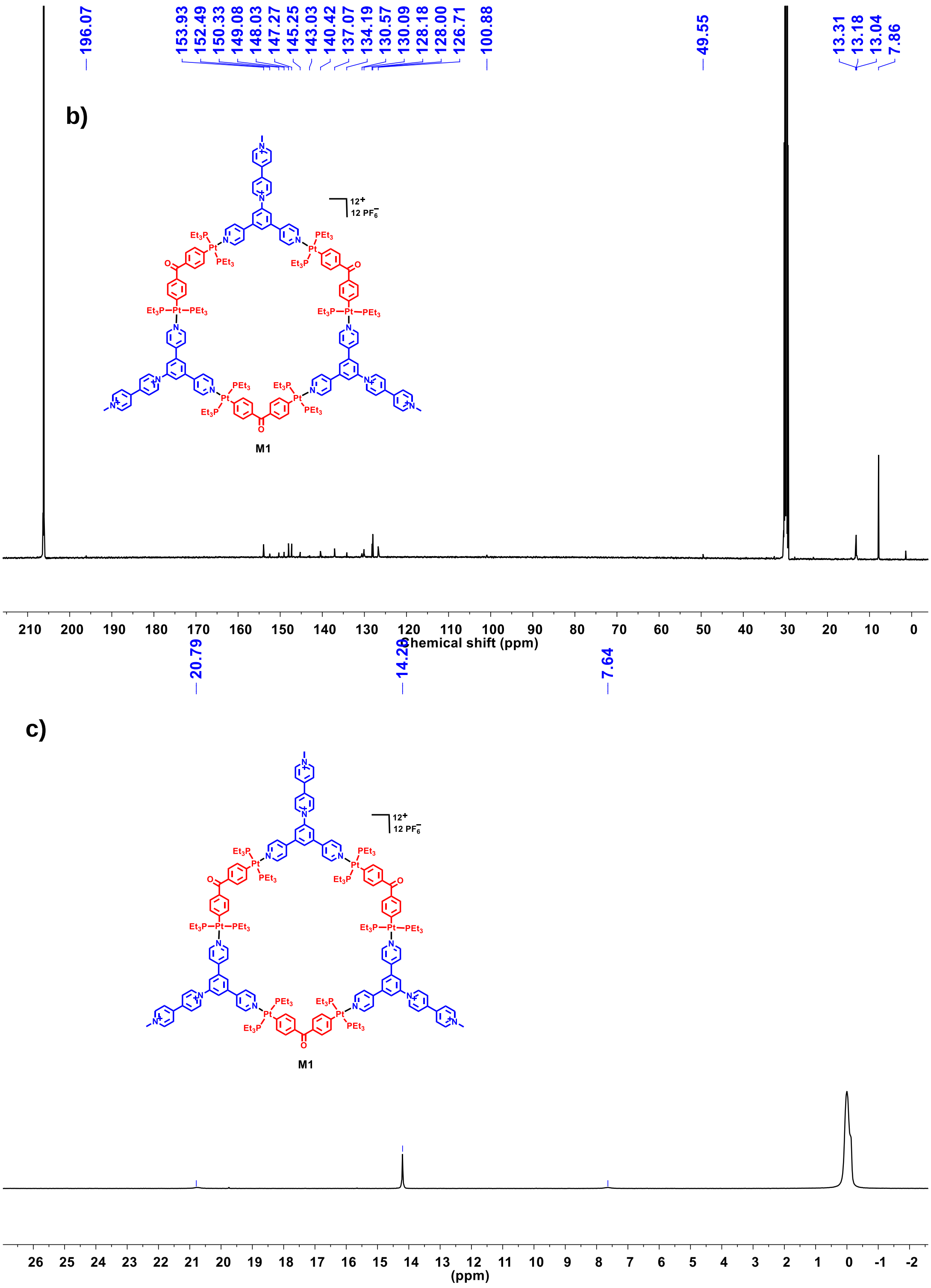

Figure S25. **a**) ^{1}H NMR spectrum (500 MHz, *d*-acetone, 298 K), **b**) ^{13}C NMR spectrum (126 MHz, *d*-acetone, 298 K), **c**) ^{31}P NMR spectrum (202 MHz, *d*-acetone, 298 K) of compound **M1**.

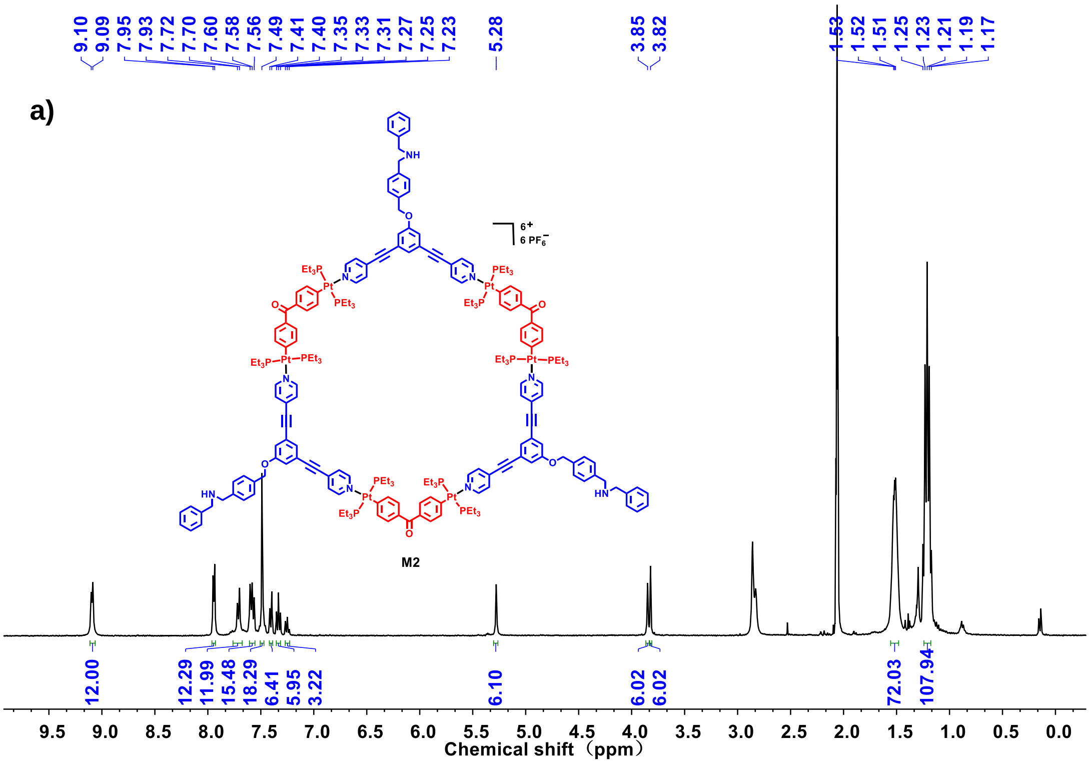
a)
9.10
9.09
7.95
7.93
7.72
7.70
7.60
7.58
7.56
7.49
7.41
7.40
7.35
7.33
7.31
7.27
7.25
7.23
5.28
3.85
3.82
1.53
1.52
1.51
1.25
1.23
1.21
1.19
1.17
12.00
12.29
11.99
15.48
18.29
6.41
5.95
3.22
6.10
6.02
6.02
72.03
107.94
6+
6 PF6−
M2
9.5 9.0 8.5 8.0 7.5 7.0 6.5 6.0 5.5 5.0 4.5 4.0 3.5 3.0 2.5 2.0 1.5 1.0 0.5 0.0
Chemical shift（ppm）

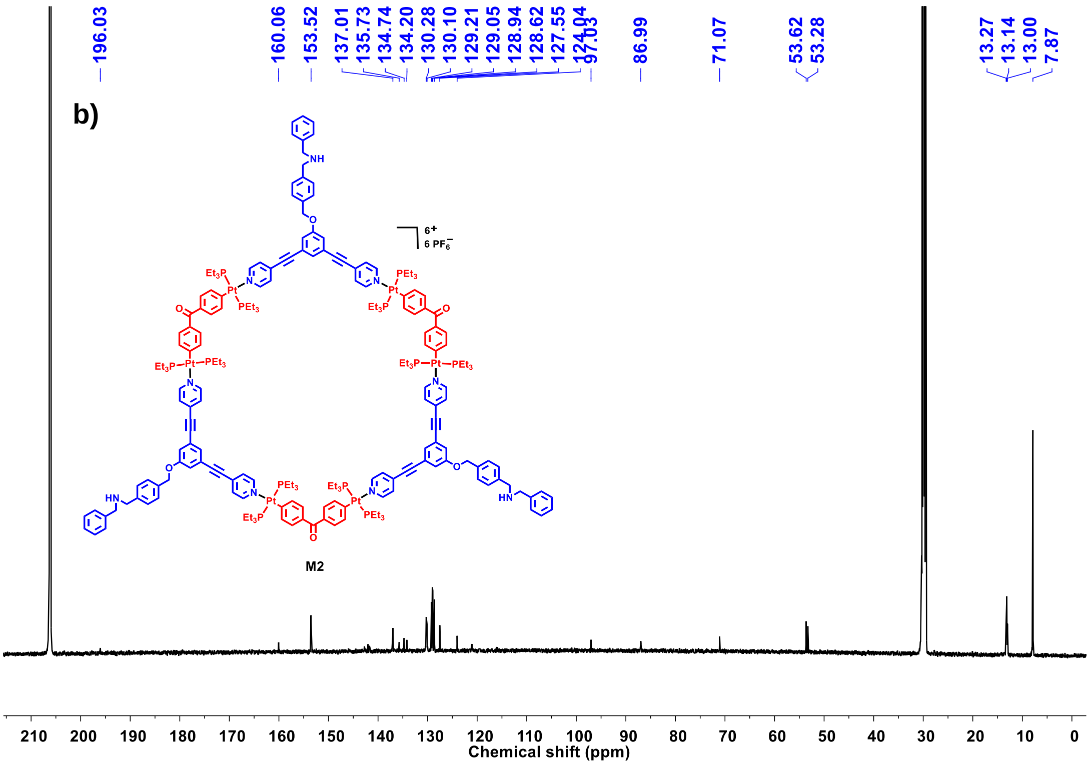
b)
196.03
160.06
153.52
137.01
135.73
134.74
134.20
130.28
130.10
129.21
129.05
128.94
128.62
127.55
124.04
97.03
86.99
71.07
53.62
53.28
13.27
13.14
13.00
7.87
6+
6 PF6−
M2
210 200 190 180 170 160 150 140 130 120 110 100 90 80 70 60 50 40 30 20 10 0
Chemical shift (ppm)

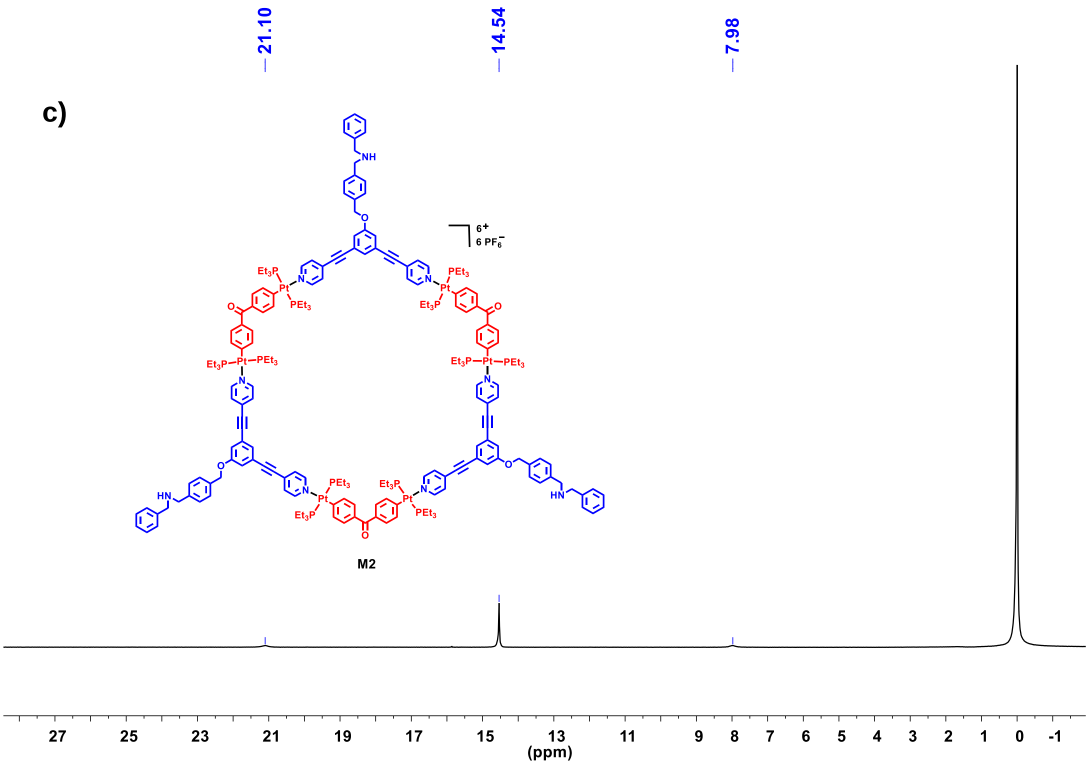

Figure S26. **a**) ^{1}H NMR spectrum (500 MHz, *d*-acetone, 298 K), **b**) ^{13}C NMR spectrum (126 MHz, *d*-acetone, 298 K), **c**) ^{31}P NMR spectrum (202 MHz, *d*-acetone, 298 K) of compound **M2**.

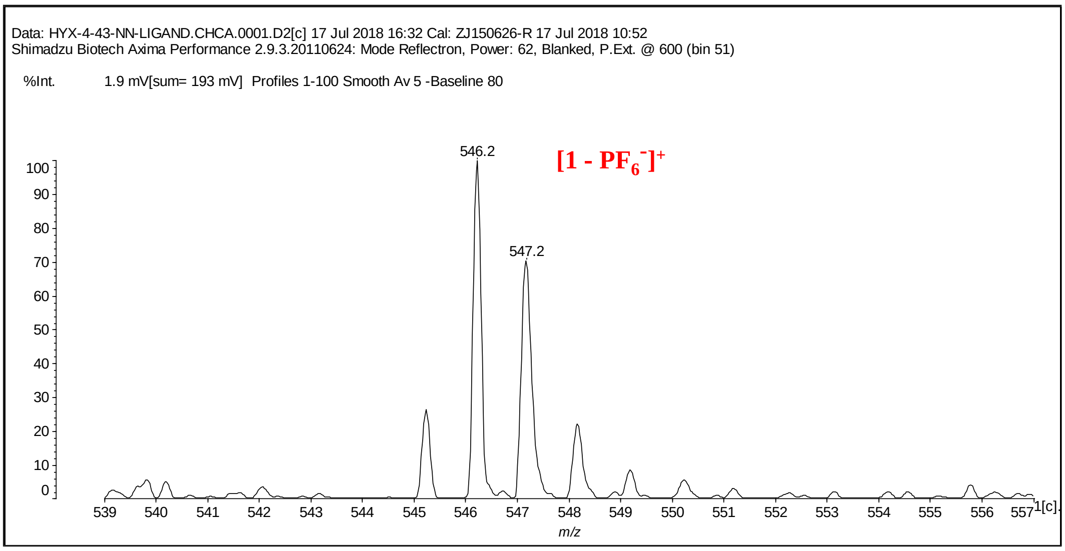

Figure S27. MALDI-TOF-MS for compound **1**: calcd for [**1** − PF_6^-]$^+$: 547.1, found: 547.2.

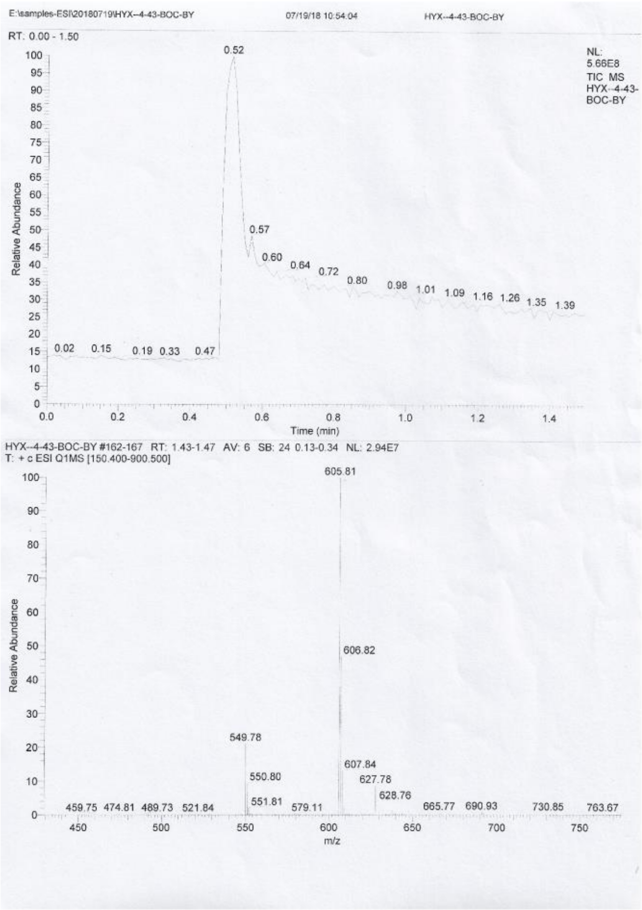

Figure S28. MS(ESI-TOF) for compound **S5**: calcd for [**S5**]$^+$: 605.74; Found: 605.81.

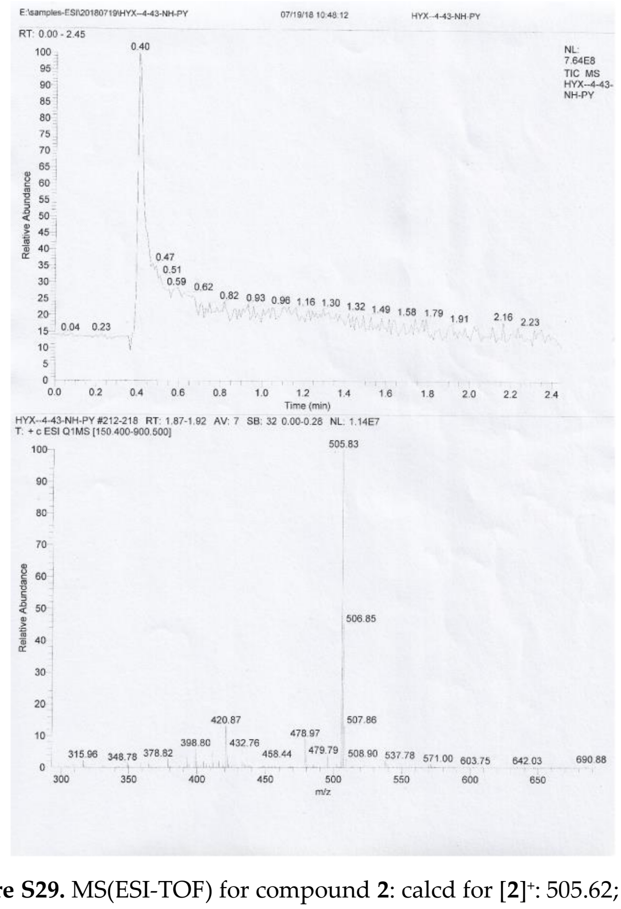

Figure S29. MS(ESI-TOF) for compound **2**: calcd for [**2**]$^+$: 505.62; Found: 505.83.

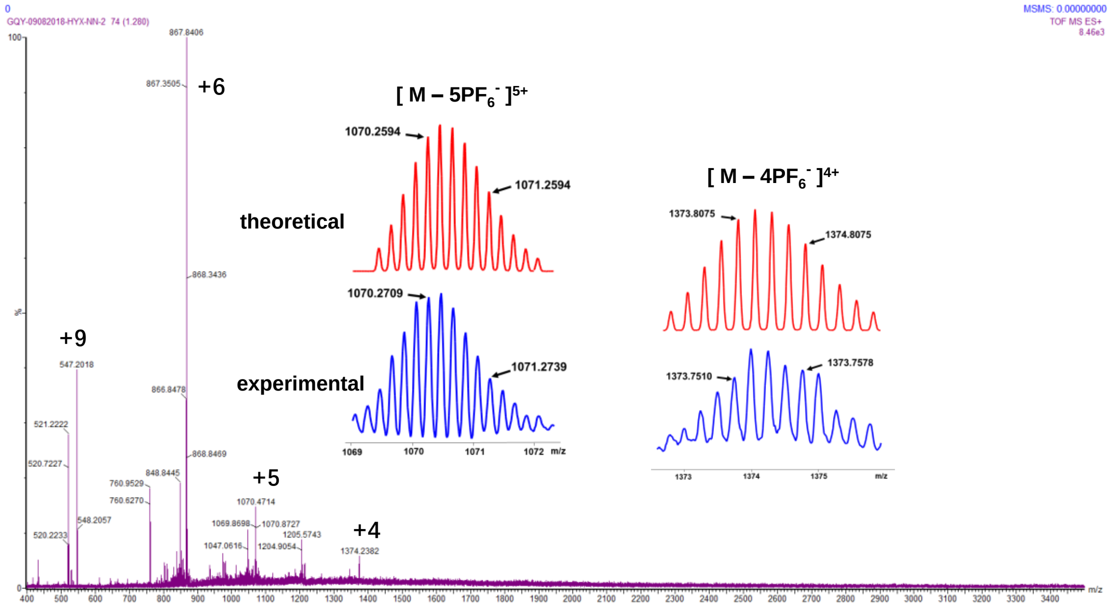

Figure S30. ESI-TOF-MS spectrum of metallacycle **M1**: *m/z* calcd for $[M - 4PF_6^-]^{4+}$: 1374.0639, found: 1373.9945; *m/z* calcd for $[M - 5PF_6^-]^{5+}$: 1070.2583, found: 1070.2709.

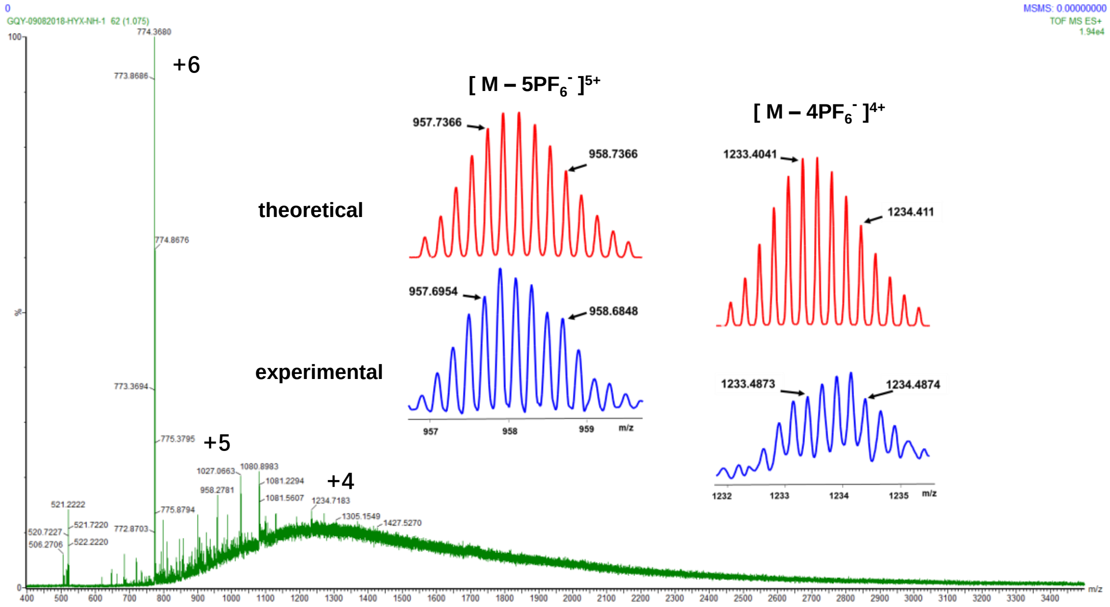

Figure S31. ESI-TOF-MS spectrum of metallacycle **M2**: *m/z* calcd for $[M - 4PF_6^-]^{4+}$: 1233.9576, found: 1233.9489; *m/z* calcd for $[M - 5PF_6^-]^{5+}$: 958.1732, found: 958.2781.

References

1. H. Yin, R. Rosas, D. Gigmes, O. Ouari, R. Wang, A. Kermagoret, D. Bardelang, *Org. Lett.* **2018**, *20*, 3187–3191.
2. S. K. Samanta, D. Moncelet, V. Briken, L. Isaacs, *J. Am. Chem. Soc*. **2016**, *138*, 14488–14496.
3. H.-B. Yang, A.M. Hawkridge, S.D. Huang, N. Das, S.D. Bunge, D.C. Muddiman, P.J. Stang, *J. Am. Chem. Soc*. **2007**, *129*, 2120–2129.
4. D. G. Amirsakis, A.M. Elizarov, M.A. Garcia-Garibay, P.T. Glink, J.F. Stoddart, A.J. White, D.J. Williams, *Angew. Chem. Int. Ed.* **2003**, *42*, 1126–1132.
5. K. Ghosh, H.-B. Yang, B.H. Northrop, M.M. Lyndon, Y.-R. Zheng, D.C. Muddiman, P.J. Stang, *J. Am. Chem. Soc.* **2008**, *130*, 5320–5334.
